# Isolating the impact of COVID-19 lockdown measures on urban air quality in Canada

**DOI:** 10.1007/s11869-021-01039-1

**Published:** 2021-05-18

**Authors:** Rabab Mashayekhi, Radenko Pavlovic, Jacinthe Racine, Michael D. Moran, Patrick M. Manseau, Annie Duhamel, Ali Katal, Jessica Miville, David Niemi, Si Jun Peng, Mourad Sassi, Debora Griffin, Chris Anthony McLinden

**Affiliations:** 1 Air Quality Policy-Issue Response Section, Canadian Meteorological Center, Environment and Climate Change Canada, Dorval, Quebec, Canada; 2 Air Quality Research Division, Environment and Climate Change Canada, Toronto, Ontario Canada

**Keywords:** COVID-19 impact, Regional air quality model, Lockdown emission scenario, Air quality observation analysis, Canadian air quality

## Abstract

We have investigated the impact of reduced emissions due to COVID-19 lockdown measures in spring 2020 on air quality in Canada’s four largest cities: Toronto, Montreal, Vancouver, and Calgary. Observed daily concentrations of NO_2_, PM_2.5_, and O_3_ during a “pre-lockdown” period (15 February–14 March 2020) and a “lockdown” period (22 March–2 May 2020), when lockdown measures were in full force everywhere in Canada, were compared to the same periods in the previous decade (2010–2019). Higher-than-usual seasonal declines in mean daily NO_2_ were observed for the pre-lockdown to lockdown periods in 2020. For PM_2.5_, Montreal was the only city with a higher-than-usual seasonal decline, whereas for O_3_ all four cities remained within the previous decadal range. In order to isolate the impact of lockdown-related emission changes from other factors such as seasonal changes in meteorology and emissions and meteorological variability, two emission scenarios were performed with the GEM-MACH air quality model. The first was a Business-As-Usual (BAU) scenario with baseline emissions and the second was a more realistic simulation with estimated COVID-19 lockdown emissions. NO_2_ surface concentrations for the COVID-19 emission scenario decreased by 31 to 34% on average relative to the BAU scenario in the four metropolitan areas. Lower decreases ranging from 6 to 17% were predicted for PM_2.5_. O_3_ surface concentrations, on the other hand, showed increases up to a maximum of 21% close to city centers versus slight decreases over the suburbs, but O_x_ (odd oxygen), like NO_2_ and PM_2.5_, decreased as expected over these cities.

**Supplementary Information:**

The online version contains supplementary material available at 10.1007/s11869-021-01039-1.

## Introduction

A novel coronavirus disease 2019 (COVID-19) was initially reported in the city of Wuhan, China, on 31 December 2019 and then began to spread around the world. On 11 March 2020, with cases of COVID-19 reported in 114 countries, the World Health Organization (WHO) declared the outbreak of this new coronavirus a pandemic (WHO 2020). To control the rapid spread of the virus, most countries were forced to adopt emergency control measures, including travel restrictions, regional lockdowns, social distancing, stay-at-home and shelter-in-place orders, and shutdowns of non-essential businesses. These measures led to significant reductions in road traffic, air travel, industrial operations, construction, and commercial business operations, which in turn reduced anthropogenic emissions of air pollutants and resulted in cleaner air quality worldwide (Bauwens et al. 2020; Liu et al. 2020; NASA 2020; Rodríguez-Urrego and Rodríguez-Urrego 2020). Such societal responses to the pandemic around the world make this crisis a natural emissions-reduction experiment that provides a unique opportunity to study anthropogenic impacts on air quality (AQ) in many countries and the potential of future air pollution controls (Henneman et al. 2017).

Many media articles appeared in the spring of 2020 reporting on satellite images released by several space agencies that revealed a marked drop in air pollutants in different regions during lockdown periods (ESA 2020a, b; NASA 2020; Schindler 2020). These articles were soon followed by scientific publications for different regions around the world. Some of these scientific studies were based on satellite observations (e.g., Bauwens et al. 2020; Liu et al. 2020; Muhammad et al. 2020; Sarfraz et al. 2020; Zhang et al. 2020), some on analysis of near-real-time (NRT) surface measurements (e.g., Rodríguez-Urrego and Rodríguez-Urrego 2020; Tobías et al. 2020; Wang et al. 2020b), and some on modeling emission scenarios (e.g., Menut et al. 2020; Sharma et al. 2020; Wang et al. 2020a). Reductions ranging from 20 to 40% have been reported in nitrogen dioxide (NO_2_) concentrations in China, the USA, Italy, Spain, France, and the UK (Berman and Ebisu 2020; Lee et al. 2020; Muhammad et al. 2020). Reductions in PM_2.5_ levels as large as 65% (Bogotá, Colombia) have been found in an analysis of 50 capital cities, many in the developing world (Rodríguez-Urrego and Rodríguez-Urrego 2020).

An important issue in the studies based on observations is the difficulty in disentangling the impact of pandemic-related emission reductions from other factors that might also contribute to an observed reduction in air pollutants. Such confounding factors include normal seasonal changes in meteorology, chemistry, and emissions, as well as anomalies in regional- and synoptic-scale meteorology compared to typical climatic patterns (Goldberg et al. 2020). For example, changes in emissions due to lockdown measures widely coincided with the transition from winter to spring in the Northern Hemisphere, thus complicating efforts to isolate the pandemic-related AQ response. Unusual variations in weather, such as extended cold or warm periods or wet or dry periods, can also affect pollutant levels (European Commission 2020; Schiermeier 2020). It is, therefore, challenging to quantify what fraction of the observed AQ improvement is linked to reductions in air pollutant emissions due to COVID-19 lockdowns and what fraction is due to the seasonal transition from winter to spring, or to the occurrence of favorable or unfavorable spring weather, or to normal seasonal variations in emissions. One common, observation-based approach is to perform an analysis of pre-lockdown versus lockdown surface or satellite measurements and compare with measurements for the same periods in past years. Emission scenario modeling with atmospheric transport models is another useful, complementary approach to quantify and isolate the impacts of emission change since actual meteorology but different emissions can be examined (e.g., Menut et al. 2020; Wang et al. 2020a).

COVID-19-related emergency measures were introduced in North America later than in China or Europe. In Canada, such measures began in mid-March 2020, and reductions in air pollutant levels have been reported in different Canadian urban areas since March 2020 (Adams 2020; Griffin et al. 2020; Rabson 2020; Xing 2020; MELCC 2020a). However, as the world’s second-largest country but with a much lower population density than China or Europe, Canada poses a particular challenge for identifying AQ impacts from COVID-19 control measures. For that reason, in this study, we have focused on large population centers where we expected the impacts to be the most pronounced. We have investigated the impact of activity reductions due to COVID-19 on NO_2_, PM_2.5_, and O_3_ levels in four of the largest urban areas in Canada: Montreal, Toronto, Calgary, and Vancouver. The first part of the study examines ground-level concentrations and compares measurements made during pre-COVID-19 vs. lockdown periods in 2020 and during the same periods from 2010 to 2019. In the second part of the study, we used the Canadian operational air quality GEM-MACH (Global Environmental Multiscale–Modelling Air-quality and Chemistry) model to quantify the impact of reduced emissions in isolation. We performed two 2020 emissions-scenario simulations: a baseline “business-as-usual” (BAU) simulation that employs “normal” emissions and serves as the counterfactual (Henneman et al. 2017), and a COVID-19 scenario that accounts for emission changes, both decreases and increases, due to lockdown measures. The “Surface observational analysis” section describes the analysis of NRT surface observations and the modeling analysis is described in the “Modeling approach” section. Analysis results are then discussed in the “Discussion” section and conclusions are provided in the “Summary and conclusions” section.

## Surface observational analysis

### Measurement data description

Hourly NRT measurements of NO_2_, PM_2.5_, and O_3_ surface concentrations for Canada were obtained from provincial and municipal air quality monitoring networks that are part of the larger National Air Pollution Surveillance (NAPS) Program (NAPS 2020; MELCC 2020b). The measurements are transmitted either directly to Environment and Climate Change Canada (ECCC) or indirectly via the US Environmental Protection Agency’s AirNow system (https://docs.airnowapi.org/). These measurements are then entered into the ECCC Verification for Air QUality Models (VAQUM) system (Gilbert et al. 2014), an evaluation tool that produces various statistical scores of model predictions vs. measurements for the ECCC operational AQ forecast system (e.g., Moran et al. 2013; Pavlovic et al. 2016). Before the NRT AQ measurements are used by VAQUM, they undergo a number of quality-control tests, including identification of exceedances of maximum concentration thresholds (200 ppbv for NO_2_, 300 μg/m^3^ for PM_2.5_, 300 ppbv for O_3_) and minimum concentration thresholds (−3 ppbv for NO_2_ and O_3_ and −3 μg/m^3^ for PM_2.5_) and detection of dubious sudden jumps and spikes (see Section S1 for more details). The NRT measurements are nevertheless considered to be preliminary and are subject to change when different agencies release final, quality-assured measurement data sets for the same period. This last step, however, can typically take over 6 months, and in the meantime, the NRT measurements provide valuable information on recent air quality conditions and on model forecast capability. However, for the period 2010–2019, which we also considered, we obtained final NAPS measurement data sets.

**Fig. 1:**
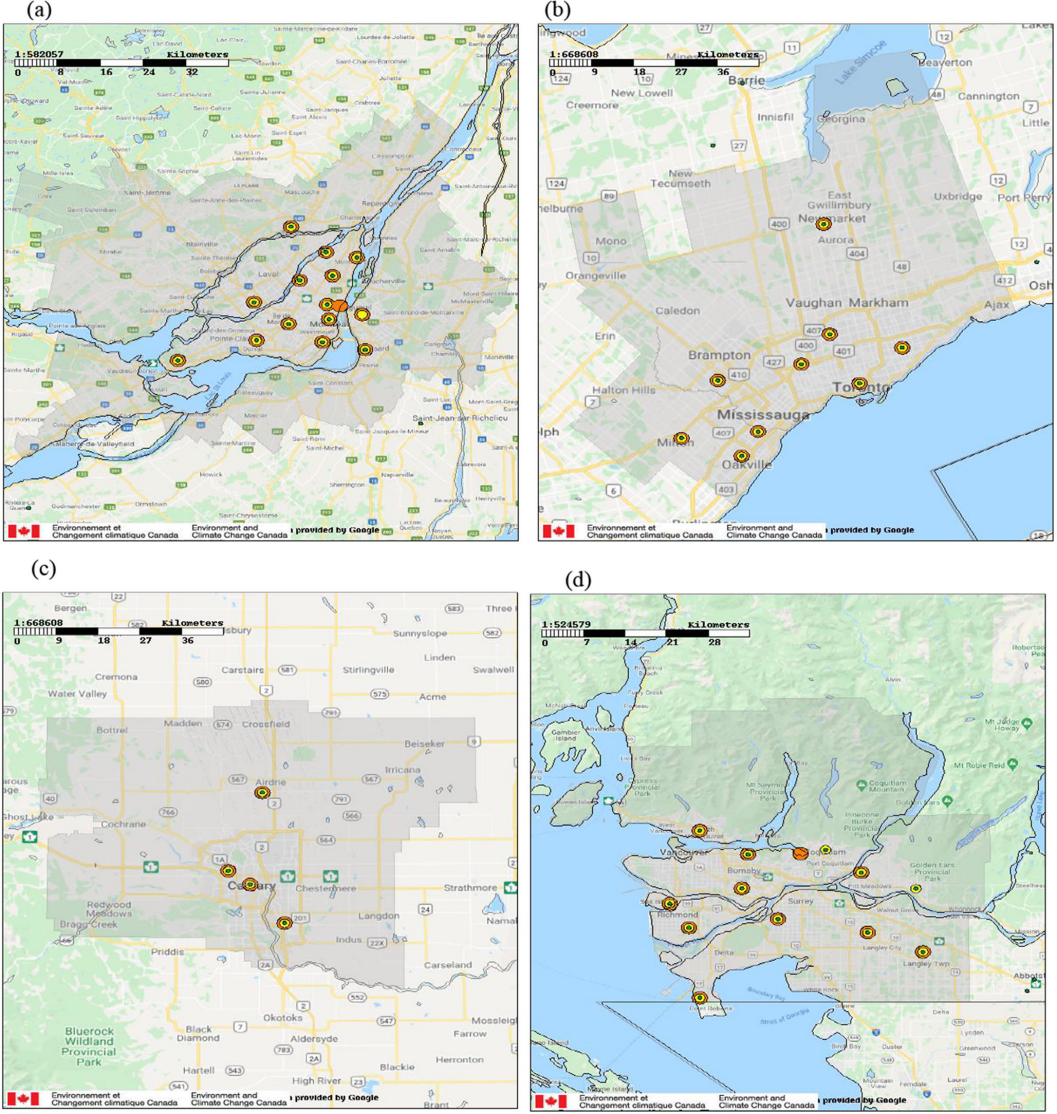
Locations of NAPS monitoring stations for NO_2_ (shown as yellow circles), PM_2.5_ (orange circles), and O_3_ (green circles) within the Canadian Census Metropolitan Areas (shaded gray regions) for (**a**) Montreal (15 stations, 4.1 million population), (**b**) Toronto (9 stations, 6.2 million population), (**c**) Calgary (4 stations, 1.6 million population), and (**d**) Vancouver (13 stations, 2.6 million population)

Measurements from sites located in the four largest Canadian Census Metropolitan Areas (CMA )—Montreal, Toronto, Calgary, and Vancouver—were considered for this analysis. A CMA consists of one or more adjacent municipalities surrounding an urban core, where the core must have a population of at least 50,000 and the entire CMA must have a total population of at least 100,000 (CMA 2011). The populations of these four CMAs range from 1.6 to 6.2 million. Maps of the four CMAs and the locations of measurement sites within each CMA are presented in Fig. 1. For the Montreal CMA, there are 14 measurement stations for NO_2_, 15 for PM_2.5_, and 13 for O_3_; for the Toronto and Calgary CMAs, there are a total of nine and four measurement stations, respectively, for all three pollutants; and for the Vancouver CMA, there are 12 measurement stations for NO_2_, 11 for PM_2.5_, and 12 for O_3_. All of these measurement sites reported at least 80% of the time (96% on average) during the 4 months from February to May 2020, the period of interest for this study. Although some other CMAs, like Ottawa-Gatineau, were also of interest, they each had fewer than four measurement sites, a small sample size that raised concerns about spatial representativeness.

### Pre-COVID versus COVID analysis

National and provincial declarations of states of emergency in Canada began in mid-March 2020 (Boire-Schwab et al. 2020); but complete lockdown measures were not in place until 18 March when the USA-Canada border was officially closed to all non-essential travel. Daily mobility data, including the “driving” and “transit” categories, which were extracted for the four CMAs from Apple mobility trends reports based on smartphone tracking (Apple Inc 2020), show a rapid decline in these activities for all four CMAs starting from mid-March (Figure S1). The end of the complete lockdown period considered in this study is 2 May 2020, when individual provinces began a gradual exit from lockdown by announcing the removal of several regional travel restrictions and the re-opening of some small businesses and provincial parks (Neustaeter 2020). Driving activity can be seen to begin to increase in the second half of April and to continue to grow throughout May (Figure S1). We have thus focused our analysis on two time periods, a pre-lockdown “normal” period from 15 February to 14 March 2020 and a lockdown period from 22 March to 2 May 2020, during which lockdown measures were in full force everywhere in Canada. The week-long period from 15 to 21 March is considered to be a transition period.

**Fig. 2:**
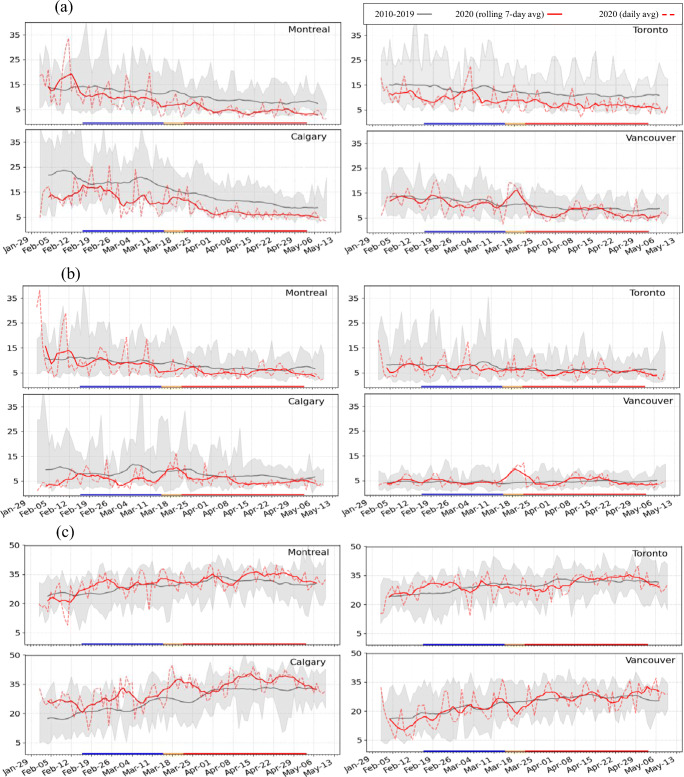
Time series of observed rolling 7-day average for hourly (a) NO_2_ (ppbv) (b) PM_2.5_ (μg m^-3^) and (c) O_3_ (ppbv) surface concentrations averaged over all monitoring stations within each city from 1 February to 13 May for year 2020 (solid red line) and for the preceding 10-year-period (2010–2019) average (gray line). The dashed red line corresponds to the daily average in 2020 and the shaded gray area presents the daily minimum and maximum values observed in the 10-year data. The blue, yellow, and red lines shown on the time axis below each plot indicate the pre-lockdown, transition to full lockdown, and full lockdown periods, respectively

In order to account for normal seasonal changes from February to May, such as increasing solar insolation and temperature, observed surface concentrations during the pre-lockdown and lockdown periods in 2020 were compared to the same periods in the previous 10 years (2010-2019). Figure 2 shows time series plots of NO_2_, PM_2.5_, and O_3_ surface concentrations for the February to May period for 2020 and 2010–2019, for the four CMAs. Seasonal decreases can be seen for both NO_2_ and PM_2.5_ whereas O_3_ shows a slight increase. The 2020 time series for mean NO_2_ volume mixing ratio (VMR) is lower than the 2010–2019 mean for all four CMAs for both the pre-lockdown and lockdown periods, suggesting that early 2020 is already an atypical period. For PM_2.5_ concentrations, on the other hand, the 2020 mean time series lies below the 2010–2019 mean time series only for Montreal and Calgary, and for O_3_ only the 2020 mean time series for Calgary is consistently higher than the 2010–2019 mean time series.

Table 1 shows overall mean values for the pre-lockdown vs. lockdown periods for 2020 and 2010–2019 for the four CMAs. The mean seasonal reduction in NO_2_ VMR (lockdown mean subtracted from pre-lockdown mean) observed for the 10-year baseline varies by city, from 19% for Toronto up to 40% for Calgary (Table 1). A similar but higher seasonal decline is observed in 2020 for each city, with reductions of 25% for Toronto, 47% for Calgary, 50% for Montreal (vs. 33%), and 33% for Vancouver (vs. 27%). One can then subtract the seasonal change in 2020 from the previous 10-year average change to estimate the impact of the COVID-induced change. These additional reductions for NO_2_ are 17% for Montreal, 6% for Toronto, 7% for Calgary, and 6% for Vancouver. However, this approach does not account for meteorological variations (anomalies from climate averages) in 2020, which can also impact observed concentrations independently of emission changes. For example, the pre-lockdown period in 2020 was also characterized by lower concentrations compared to the same period in the baseline average (Table 1). Mean NO_2_ VMR values during the pre-lockdown period in 2020 were lower than the corresponding values for the previous 10-year period for each CMA by 26% for Montreal, 31% for Toronto, 31% for Calgary, and 11% for Vancouver. Goldberg et al. (2020) noted that meteorological patterns favored lower NO_2_ levels in spring 2020 compared to spring 2019. In particular, snow cover extent and depth were much reduced in early March in 2020 vs. 2019, which would lead to increased vertical mixing and hence could be an important factor for Montreal, Toronto, and Calgary (Vancouver has a milder climate).

**Table 1:** Mean hourly observed NO_2_, PM_2.5_, and O_3_ concentrations averaged over the pre-lockdown and lockdown periods in 2020 and 2010–2019 for the four CMAs

		NO_2_ (ppbv)			PM_2.5_ (μg m^−3^)			O_3_ (ppbv)		
		Pre-lockdown	Lockdown	% change^2^	Pre-lockdown	Lockdown	% change^2^	Pre-lockdown	Lockdown	% change^2^
Montreal (14 stations for NO_2_, 15 for PM_2.5_, and 13 for O_3_)	2010–2019	12.7	8.5	−33.1	9.8	6.7	−31.6	27.5	31.7	15.3
	2020	9.4	4.7	−50.0	8.5	5.5	−35.3	30.0	33.3	11.0
	% change^1^	−26.0	−44.7		−13.3	−17.9		9.1	5.0	
Toronto (9 stations for NO_2_, PM_2.5_, and O_3_)	2010–2019	13.4	10.9	−18.7	7.5	5.9	−21.3	28.1	31.7	12.8
	2020	9.3	7.0	−24.7	6.8	5.6	−17.6	29.9	31.7	6.0
	% change ^1^	−30.6	−35.8		−9.3	−5.1		6.4	0.0	
Calgary (4 stations for NO_2_, PM_2.5_, and O_3_)	2010–2019	18.9	11.4	−39.7	8.7	7.2	−17.2	23.1	31.4	35.9
	2020	13.0	6.9	−46.9	5.6	5.1	−8.9	27.3	36.1	32.2
	% change^1^	−31.2	−39.5		−35.6	−29.2		18.2	15.0	
Vancouver (12 stations for NO_2_, 11 for PM_2.5_, and 12 for O_3_)	2010–2019	11.8	8.6	−27.1	4.3	4.5	4.7	21.1	27.1	28.4
	2020	10.5	7.0	−33.3	4.3	4.6	7.0	21.4	27.6	29.0
	% change^1^	−11.0	−18.6		0.0	2.2		1.4	1.8	

^1^% change in 2020 compared to 2010–2019

^2^% change in lockdown period compared to pre-lockdown period

The differences between the seasonal changes in PM_2.5_ surface concentration for 2020 vs. 2010–2019 for the four CMAs are less pronounced than those for NO_2_ (Table 1). The observed PM_2.5_ seasonal decreases in 2020 in Toronto and Calgary were smaller than the mean seasonal decreases for 2010–2019, whereas Montreal had a 4% greater reduction in 2020 than the 2010–2019 average. Vancouver, by contrast, had a 7% increase in PM_2.5_ in 2020 from the pre-lockdown to lockdown period, slightly higher than the 5% increase for the 2010–2019 baseline. For O_3_, which has seasonal springtime increases for the 10-year baseline for all four CMAs (15% in Montreal, 13% in Toronto, 36% in Calgary, and 28% in Vancouver), the seasonal increases in 2020 were smaller for Montreal (−4%), Toronto (−7%), and Calgary (−4%) and was essentially unchanged for Vancouver. It is thus difficult to discern an obvious COVID-19 signal for either PM_2.5_ or O_3_.

**Fig. 3:**
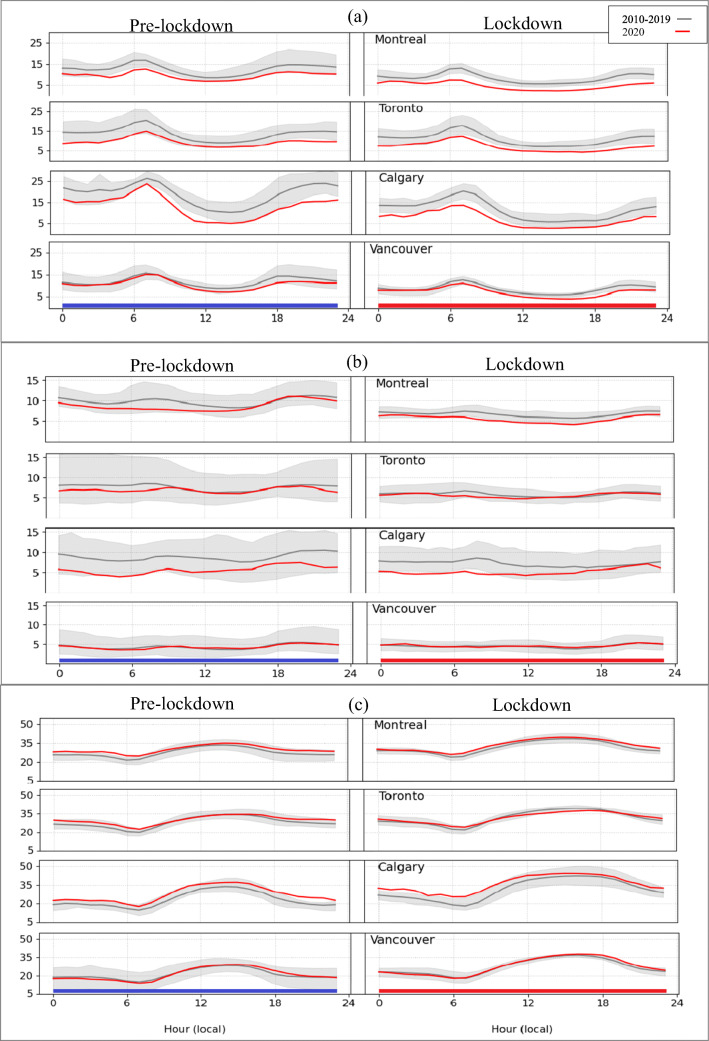
Mean diurnal time series for observed hourly (a) NO_2_ (ppbv) (b) PM_2.5_ (μg m^-3^) and (c) O_3_ (ppbv) concentrations during pre-lockdown (left panels) and lockdown period (right panels) for four major metropolitan areas in Canada. The red line shows the mean values for 2020 and the black line shows the mean values for 2010–2019 with the ranges for individual years shaded in gray

It is also of interest to look at pre-lockdown and lockdown mean diurnal time series for each city for 2020 vs. 2010–2019 (Fig. 3). In 2020, the NO_2_ mean diurnal time series were lower than the 10-year average during both the pre-lockdown and lockdown periods for all four cities (Fig. 3(a)). Moreover, the 2020 NO_2_ lockdown diurnal time series for Toronto, Montreal, and daytime Vancouver lie below the shaded gray regions, indicating that the 2020 values are lower than the minimum values observed for any year in the previous 10 years. At the same time, the peak values for Toronto, Montreal, and Calgary during the lockdown period, which occur during morning rush hour in each city, lie below the 2010–2019 range. These lower values could be linked in part to COVID-19 lockdown measures. The PM_2.5_ diurnal time series also show lower concentrations than the 10-year average during both the pre-lockdown and lockdown periods for Montreal, Calgary, and, to some extent, Toronto, whereas the 2020 PM_2.5_ diurnal time series for Vancouver are very close to those for the 2010–2019 period (Fig. 3(b)). Lastly, the differences in the mean diurnal time series between 2020 and the 2010–2019 baseline averages for O_3_ are relatively small for all four cities (Fig. 3(c)).

The results of this simple statistical analysis confirm the difficulty of trying to quantify the impact of COVID-induced reductions in pollutant levels without considering meteorological anomalies. Short-term variations in meteorological fields can also have large short-term impacts on observed pollutant concentrations. For example, elevated peaks of NO_2_ and PM_2.5_ were observed in spring 2020 in Vancouver centered on 18 March and in mid-April (Fig. 2). A meteorological analysis (Figure S5) found that Vancouver was under a positive geopotential anomaly at 500 hPa and a positive surface pressure anomaly during both of these periods (IRI 2020). This means that for both peaks, this region was situated under a stable, high-pressure system, which is favorable for high pollutant concentrations even with reduced emissions. Wang et al. (2020b) also noted the importance of meteorological variations during the COVID-19 lockdown period.

It should also be noted that this observation-based analysis did not consider inter-annual changes in Canadian anthropogenic emissions, although marked decreases in emissions have occurred over the 2010–2017 period for many pollutants (CESI 2020). The impact of wildfire emissions was also not considered, as wildfires are not typically a significant PM_2.5_ source in early spring in Canada (e.g., Munoz-Alpizar et al. 2017).

Nevertheless, the analyses presented in this section suggest that a higher-than-usual change in observed pollutant surface concentrations occurred in the four largest Canadian urban areas during the COVID-19 lockdown period, especially for NO_2_ and, to a lesser extent, PM_2.5_. Further quantification of the impact of COVID-19-related measures, however, is limited by the difficulty in accounting for the simultaneous impact of seasonal and inter-annual variations in meteorology and emissions.

## Modeling approach

This section describes an additional analysis of the impact of COVID-19 measures for the four Canadian CMAs, where an AQ model was used to simulate two 2020 emission scenarios. One was a BAU scenario that employed “normal” emissions unaffected by any COVID-19 measures. The other was a COVID-19 scenario that accounted for emission changes, both decreases and increases, due to lockdown measures. These two scenarios were simulated using the same 2020 meteorology and usual seasonal changes in emissions, which allowed the impact of the unusual, COVID-19-related emission changes to be isolated and quantified.

### Model and simulation description

The air quality model used in this study was version 3.0 of the ECCC Global Environmental Multiscale–Modelling Air-quality and Chemistry (GEM-MACH) model. GEM-MACH is an in-line, one- or two-way-coupled chemical transport model (CTM) with a detailed representation of atmospheric chemistry, including emissions, dispersion, and removal processes, that is embedded within the GEM model, ECCC’s operational global and regional numerical weather prediction (NWP) model (Caron et al. 2015; Charron et al. 2012; Côté et al. 1998b; Côté et al. 1998a). The GEM-MACH model has been used operationally by ECCC for regional air quality forecasting since 2009 (Moran et al. 2010, 2013; Pavlovic et al. 2016), and version 3.0 has been operational since July 2019 (Moran et al. 2019a).

GEM-MACH is a multi-phase, multi-pollutant CTM that considers the interactions of gas-, aqueous-, and particle-phase chemical components. To model gas-phase chemistry, it employs an updated version of the ADOM-2 gas-phase chemical mechanism with 42 species and 114 reactions (Stockwell and Lurmann 1989; Stroud et al. 2008; Venkatram et al. 1992). The aqueous-phase chemistry mechanism is based on an updated version of the ADOM mechanism with 13 species and 25 reactions (Fung et al. 1991; Gong et al. 2006). The chemical composition of particulate matter (PM) is represented using eight chemical components: sulfate; nitrate; ammonium; elemental carbon; primary organic matter; secondary organic matter; crustal material; and sea salt. The treatment of aerosol-phase dynamics includes parameterizations of nucleation, condensation, coagulation, dry deposition, aerosol-cloud interactions, and cloud scavenging (Gong et al. 2003); inorganic aerosol thermodynamics, cloud processing, and secondary organic aerosol chemistry are also considered (Gong et al. 2015; Makar et al. 2003; Stroud et al. 2011). A simplified, two-bin sectional PM size distribution with Stokes diameter size bins of 0–2.5 and 2.5–10 μm to represent fine PM (PM_2.5_) and the coarse fraction of PM_10_, respectively, is used by the operational version of GEM-MACH to reduce computational expense.

Anthropogenic emissions of 18 gas-phase species and 12 size-bin-specific PM chemical components are needed by the model. Plume-rise and plume-spread calculations are performed at each model time step for large stationary point sources to determine the model vertical layers into which point-source emissions are injected. Biogenic emissions and sea-salt emissions are also calculated in the model at each time step based on meteorological fields forecast by the model. Algorithms from version 3.09 of the BEIS biogenic emissions model (Hanna et al. 2005) and from the sea-salt emission scheme of Gong et al. (2003) are used to estimate these natural emissions. The anthropogenic emissions used in this study are described in more detail in the next section.

**Fig. 4:**
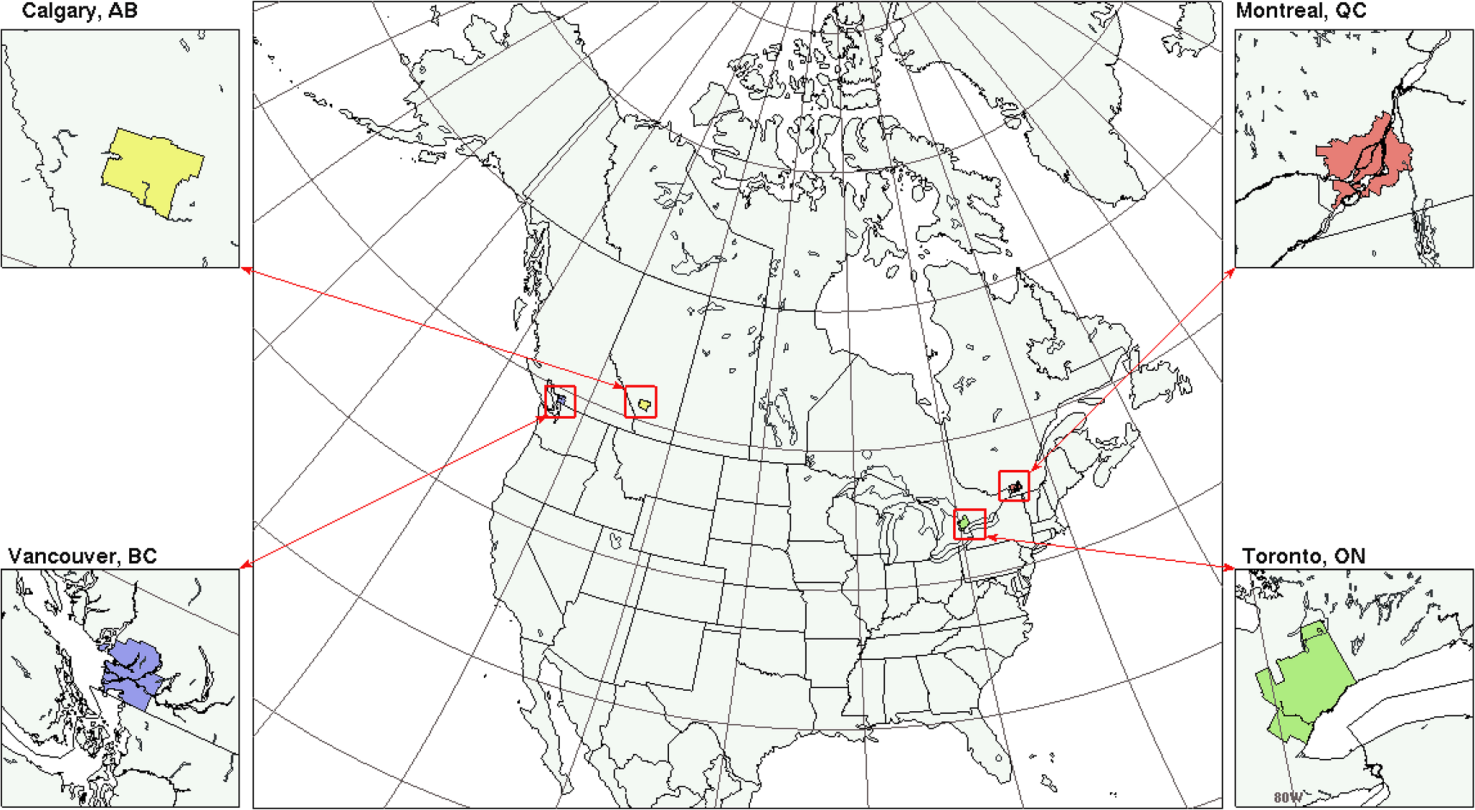
Map of the GEM-MACH domain. The boxes show the locations and spatial extent of the Census Metropolitan Areas for the four major Canadian cities considered in this study

For operational regional forecasting, GEM-MACH is run in a one-way-coupled mode twice a day at 00 and 12 UTC to produce 72-h forecasts of three air pollutants (NO_2_, PM_2.5_, and O_3_) over North America. The forecast domain covers most of Canada, the continental US, and northern Mexico (Fig. 4) on a latitude-longitude map projection and a horizontal grid with 10-km grid spacing. In the vertical, a hybrid sigma-pressure coordinate is used with a Charney-Phillips staggered vertical grid, with 84 momentum full levels and thermodynamic half levels from the Earth’s surface up to 0.1 hPa. The lowest three momentum levels are located at 20 m, 60 m, and 115 m AGL and the first three thermodynamic levels are located at 10 m, 40 m, and 85 m. Chemical tracers are assigned to thermodynamic levels.

The GEM-MACH regional forecast grid is a subgrid of a larger 10-km forecast grid used by the regional version of the GEM NWP model. Hourly meteorological boundary conditions are provided from 72-h GEM operational meteorological forecasts. Predetermined, spatially varying seasonal chemical lateral boundary conditions are based on a 1-year simulation of the MOZART4 (Model for OZone and Related chemical Tracers) global CTM, which includes a detailed treatment of tropospheric inorganic chemistry and some organic species (Pendlebury et al. 2018). The regional version of GEM uses an integration time step of 300 s while the chemistry module of GEM-MACH employs an integration time step of 900 s to reduce computational expense. Note, though, that operator splitting is employed to integrate the GEM-MACH chemistry module and some processes such as gas-phase chemistry may be solved using much smaller time steps.

GEM-MACH performance has been evaluated in a number of ways against surface AQ measurements (Chen et al. 2019; Moran et al. 2018a, 2019a, b; Pavlovic et al. 2016; Robichaud et al. 2016; Stroud et al. 2020; Zhao et al. 2019) and against peer models (Im et al. 2015b; Im et al. 2015a; Wang et al. 2015) for different model versions and time periods. To best align with the present study, Table S2 summarizes objective scores for NO_2_, O_3_, and PM_2.5_ for recent periods in the summer of 2019 and winter of 2020 for the same model version (v3.0) and same Canadian projected 2020 emissions inventory as used in the present study. Hourly observations from AQ measurement stations in western Canada (including Vancouver and Calgary) and eastern Canada (including Toronto and Montreal) were considered separately. Mean biases for NO_2_ were small and negative in the winter for western and eastern Canada (−0.8 and −0.5 ppbv, respectively) and small and positive in the summer (0.1 and 0.2 ppbv). O_3_ and PM_2.5_ hourly forecasts tended to have a negative mean bias in both seasons, with a range of −5.7 to 0.3 ppbv for O_3_ and −2.8 to 0.3 μg m^−3^ for PM_2.5_.

The two scenario simulations performed in this study employed the operational configuration of GEM-MACH. The BAU-scenario simulation spanned over the period from 1 February to 11 May 2020 while the simulation period for the COVID-19 scenario was shorter and started on 15 March (the beginning of the transition period). Each GEM-MACH integration began at 00 UTC and ran for 24 h. The meteorological initialization for each integration used a new meteorological analysis from the regional configuration of the GEM operational NWP model. The initial chemical tracer fields for the first simulation of the BAU scenario were obtained from the operational GEM-MACH regional forecast for 31 January 2020. For subsequent BAU runs, the initial chemical tracer fields came from the final forecast fields of the previous GEM-MACH BAU run, and the initial chemical tracer fields used for the first simulation of the COVID-19 scenario came from the final forecast fields of the 14 March 2020 BAU-scenario run.

### Emission scenarios

Annual Canadian anthropogenic emissions of eight common air pollutants (SO_2_, NO_*x*_, VOC, CO, NH_3_, PM_2.5_, PM_10_, TSP) are tabulated by province and source type in ECCC’s comprehensive Canadian Air Pollutant Emissions Inventory (APEI 2020). The Canadian anthropogenic emissions used for the BAU scenario are based on a projected 2020 national emissions inventory that was generated by ECCC for policy studies in late 2017; the projection base-year inventory was the 2015 APEI. The projected emissions estimates account for projected changes in population, economic activity, and energy use over the 5 years, from 2015 to 2020, as well as the implementation over this period of already-legislated air pollution control measures and expected facility openings or closures. However, no additional air pollution control measures that were not already mandated by the end of 2017 to come into effect by the end of 2020 were considered in the projection, hence the BAU name.

Table 2 summarizes the BAU baseline emissions of three pollutants (NO_*x*_, VOC, PM_2.5_) in each of the four CMAs during the 42-day spring lockdown period. To calculate the total emissions in each city, GEM-MACH gridded emissions were summed for the grid cells contained within the CMA boundary of each city. This table lists the main emission source categories considered in this study, namely industry, road transportation, air traffic, residential heating, and other sources. The transportation sector, which in urban areas is dominated by emissions from on-road vehicles, is the major source of nitrogen oxides, accounting for 40 to 47% of total NO_*x*_ emissions. This sector is also the second-largest source of fine particulate matter, contributing to 33% of total PM_2.5_ in Toronto, around 25% in Montreal and Vancouver and 18% in Calgary. More than half of the total PM_2.5_ emissions in all four cities comes from the industrial sector. In particular, the dominant source (75%) of PM_2.5_ emissions in the Calgary CMA is from the upstream oil and gas industry. The industrial sector also contributes more than one-third of total volatile organic compound (VOC) emissions in the four urban areas and more than half in Calgary. The other major source of VOCs is general solvent use, which is included in the “other” category in this table. Although the contribution of aircraft LTO (landing and takeoff) emissions to total urban air pollutant emissions is relatively small (ranging from 1 to 3%), its regional impact on local air quality can be significant as it is spatially localized over a small area near the airport. Lastly, the residential heating sector contributes as much as 10% of NO_*x*_ emissions in Toronto and 20% of total PM_2.5_ emissions in Montreal (where residential wood combustion is the highest of the four cities).

**Table 2:** Total NO_x_, VOC, and PM_2.5_ emissions (tons) by source sector in each CMA during the lockdown period (22 March–2 May 2020), under the business-as-usual (BAU) scenario. The percentage values in the table header indicate the estimated change for each sector that were used to build a “lockdown” emission scenario. The percentage values in the total column indicate the overall impact of the COVID-19 scenario reductions for each city and pollutant.

CMA	Species	Industry	Road Transportation	Air Traffic	Residential Heating	Others	Total
		(−30%)	(−60%)	(−80%)	(+20%)	No change	
Montreal	NO_*x*_	1,155	1,708	75	123	848	3,909 (−36%)
	VOC	2,265	456	19	462	2,966	6,168 (−14%)
	PM_2.5_	1,169	641	2	464	141	2,417 (−27%)
Toronto	NO_*x*_	1,086	2,548	193	603	1,624	6,054 (−31%)
	VOC	3,088	693	45	123	5,272	9,221 (−15%)
	PM_2.5_	2,082	1,251	3	173	259	3,768 (−36%)
Calgary	NO_*x*_	1,060	1,563	66	200	873	3,762 (−34%)
	VOC	1,719	401	24	13	1,710	3,867 (−20%)
	PM_2.5_	1,928	459	2	32	127	2,548 (−33%)
Vancouver	NO_*x*_	745	1,402	98	110	639	2,994 (−37%)
	VOC	1,964	492	35	69	2,420	4,980 (−18%)
	PM_2.5_	626	268	3	89	82	1,068 (−31%)

To construct the COVID-19 emission scenario, the 2020 BAU baseline emissions inventory for Canada was modified to account for changes in human activities in response to restrictions during the lockdown period. Since the level of activity restrictions was similar for all regions in Canada due to the coordinated, nationwide measures implemented across the country during the lockdown period in spring 2020, we assumed a uniform, nationwide emission change factor. To quantify the reduction in traffic, we used an estimate of daily “driving” activities provided by Apple Inc. (Apple Inc 2020). A reduction in traffic activity ranging from 50 to 65% was observed for the four cities over the lockdown period (Figure S1). A complementary analysis for three of the cities that shows similar reductions can be found in Figure S3. An analysis of available vehicle traffic-count data for major roads obtained for each of the cities showed similar reductions in the total number of cars (Figure S4 shows the Calgary analysis). We thus applied a 60% decrease to on-road BAU emissions nationwide in both urban and rural areas to estimate the corresponding lockdown emissions for this sector since residents everywhere were asked to stay at home except for essential travel. Aviation activity was also strongly impacted by the lockdown: total commercial airline flights in Canada were 79% lower in April 2020 compared to April 2019 (Statistics Canada 2020). To account for this decrease, we assumed an 80% reduction in emissions from aircraft LTOs nationwide for the lockdown period.

In addition to the Apple traffic activity data, we also used time series data for Canada obtained from the Google Community Mobility Reports (Google 2020) on time spent in different categorized community places such as workplaces, residences, parks, and transit stations. While these data showed a marked decrease in time spent in most community spaces for the lockdown period, they also showed roughly a 20% increase in time spent in ‘residential’ spaces for the four cities (Figure S2). Accordingly, we applied a 20% increase to residential emissions nationwide for the lockdown period to reflect such impacts as increased home heating requirements.

The estimated reduction factors for the other sectors are based on those used in similar emission scenario studies for Europe (EU 2020; Menut et al. 2020) and our best estimates. Emissions from the industrial sector were assumed to decrease uniformly by 30% nationwide (note from Figure S2 that visits to or time spent in workplaces in the provinces containing the four CMAs decreased by 50 to 60% during the lockdown), while emissions from other sectors, such as agriculture, rail, and marine, were assumed to remain unchanged. It should be noted that this is only an illustrative scenario representing one possible case and is subject to many uncertainties. More accurate Canadian emissions estimates for early 2020 may be available in the future when the 2020 APEI is released (but only annual emissions are reported).

As shown in Fig. 4, the GEM-MACH domain also included the continental US, Alaska, and northern Mexico. The US emissions used for the two scenarios came from a projected 2017 US National Emissions Inventory (NEI) that was based on the 2011 US NEI. This 2017 inventory was obtained from version 6.3 of the US Environmental Protection Agency (EPA) 2011 Air Emissions Modeling Platform for policy development applications (Moran et al. 2018b; U.S. EPA 2016). Mexican emissions for the two scenarios were based on the 2008 Mexican National Emissions Inventory, which was also obtained from the EPA 2016 Air Emissions Modeling Platform (U.S. EPA 2016).

The gridded emissions used by GEM-MACH for the USA and Mexico were assumed to remain the same in both scenarios. This choice was partly made out of necessity because the initial US response to the COVID-19 pandemic was very complicated and varied greatly in time and by state, county, and even city (e.g., Berman and Ebisu 2020; Goldberg et al. 2020). Trying to represent the mixed impact of full, partial, and no lockdowns was beyond the scope of this project. However, our analysis of a sensitivity test for the COVID-19 scenario that used a different US inventory (projected 2028 NEI) showed that the change of US emissions had a small impact on the modeled concentrations for the four major Canadian cities considered in this study (Figure S6).

Version 3.7 of the Sparse Matrix Operator Kernel Emissions (SMOKE) tool (UNC 2014), a widely used emissions processing system, was used to process the three national anthropogenic emissions inventories to generate model-ready hourly emissions from major point sources and surface sources. Major point sources are those large individual facilities (with smokestack height greater than 15 m) for which plume rise is calculated in the GEM-MACH model. The hourly gridded emissions were generated for a representative week of each month, and so they vary by hour of the day, day of the week, and month of the year. These emissions thus account for expected temporal variations due to such factors as morning and afternoon rush hours, weekday-weekend differences, and seasonal changes in emissions due to change in space-heating and air-conditioning loads and summer vacations.

### Modeling results

**Fig. 5:**
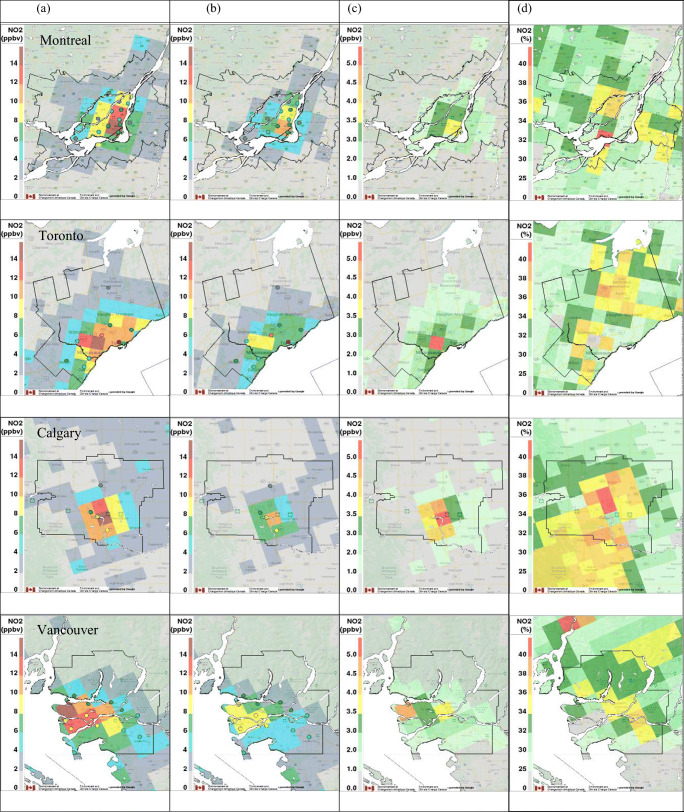
Mean hourly NO_2_ surface volume mixing ratio (ppbv) gridded fields for the “full lockdown” period (22 March–2 May 2020) predicted by the model for the four major metropolitan areas for (a) BAU scenario, (b) COVID lockdown scenario, (c) scenario difference (BAU–COVID), and (d) relative percentage difference ((BAU–COVID)/BAU). The four rows from top to bottom correspond to Montreal, Toronto, Calgary, and Vancouver. Colored circles represent the locations and mean observed NO_2_ concentrations at each monitor and the thin black outlines indicate CMA boundaries.

**Fig. 6:**
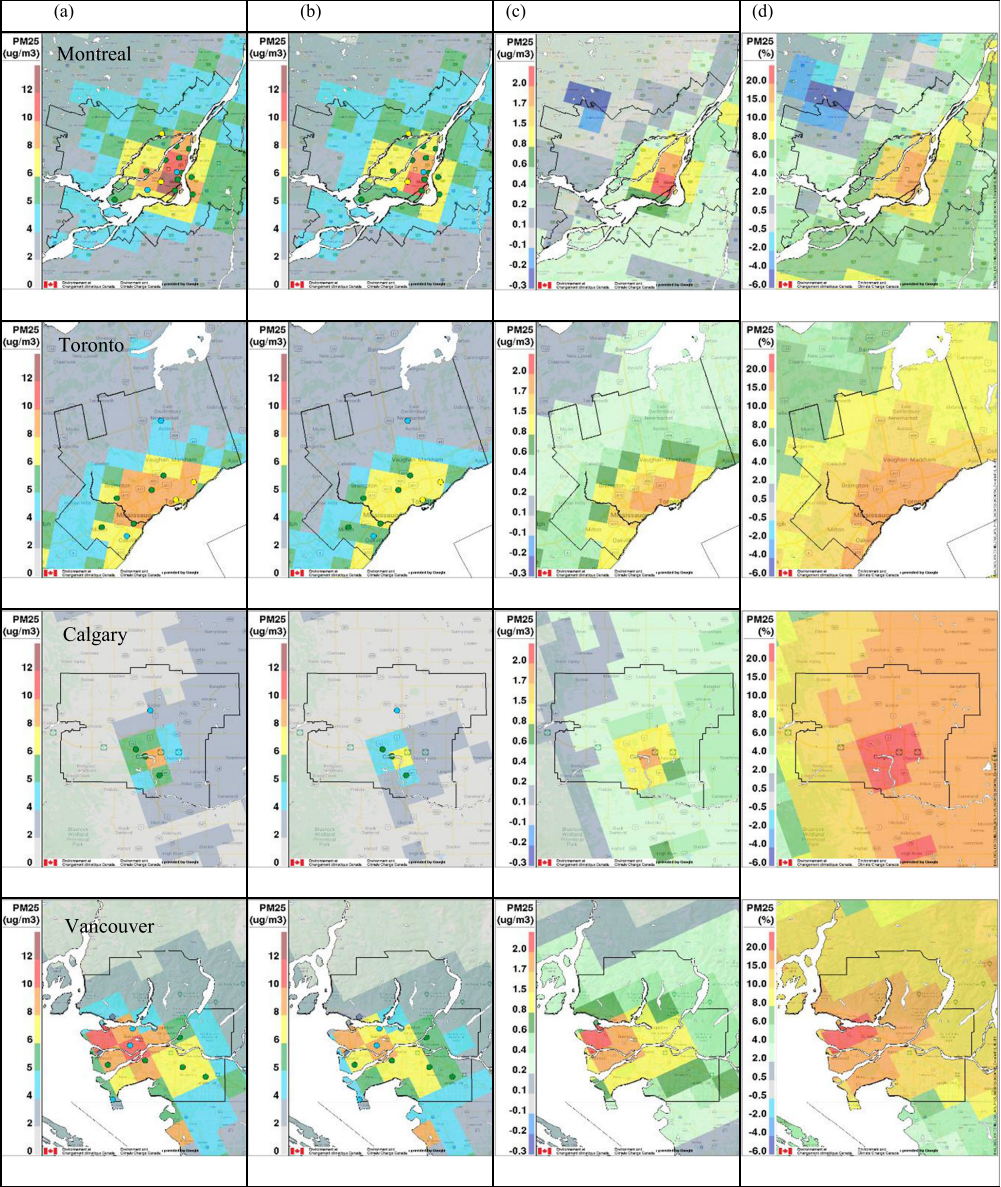
The same as Fig. 5 but for PM_2.5_ (μg m^−3^)

**Fig. 7:**
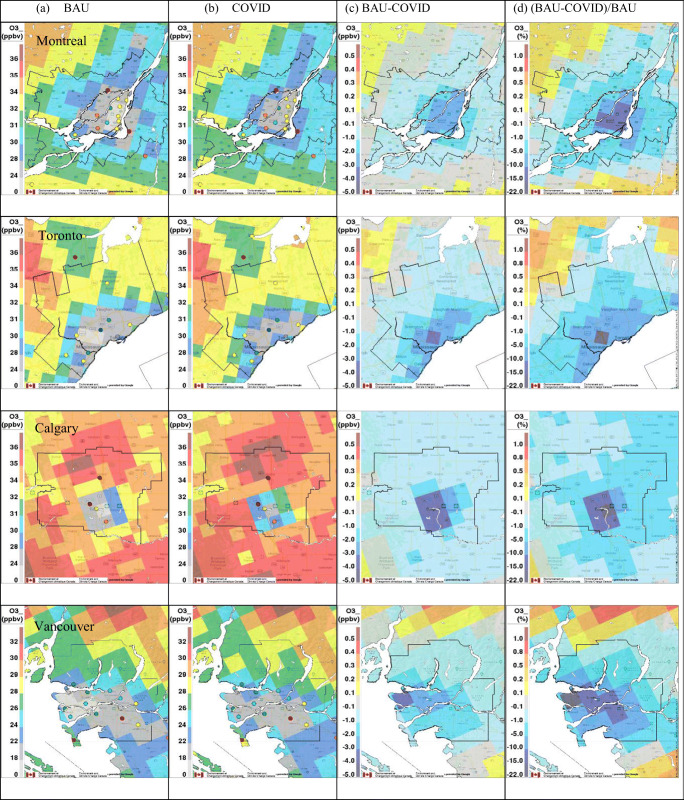
The same as Fig. 5 but for O_3_ (ppbv)

Spatial distributions of mean hourly NO_2_, PM_2.5_, and O_3_ concentrations during the lockdown period (22 March–2 May 2020) predicted by GEM-MACH for the BAU and COVID-19 scenarios are shown in Figs .5 to 7 for each major metropolitan area in the two left-side panels. Mean measured hourly concentrations are also shown by overlaying colored circles on the predicted concentration fields at the station locations. In general, better agreement with observations is seen for the COVID-19 scenario for all three pollutants as compared to the BAU scenario. The predicted mean NO_2_ and PM_2.5_ levels for the BAU scenario are higher overall than the observed mean values almost everywhere, with the exception of two stations located near downtown Toronto for NO_2_. The highest NO_2_ and PM_2.5_ concentrations predicted by the model are mainly distributed near the city centers or the intersections of major highways. The spatial patterns predicted for the COVID-19 scenario are very similar, but, consistent with the emission reduction, the magnitudes of modeled NO_2_ and PM_2.5_ levels for the COVID-19 scenario are generally lower than those predicted for the BAU scenario. By contrast, O_3_ VMR shows a slight increase for the COVID-19 scenario compared to the BAU scenario (Fig. 7), likely as a result of reduced O_3_ titration by NO due to reduced NO_*x*_ emissions.

Maps of the actual and relative difference fields between the two emission scenarios are also shown in Figs. 5–7. In Toronto, the maximum NO_2_ VMR, which corresponds to a grid cell that is located near Canada’s busiest airport and includes important highway intersections, dropped by 41% (from 14.5 to 9.3 ppbv) in the COVID scenario compared to the BAU reference run (Fig. 5). Similar but smaller reductions, ranging from 26% in Montreal (from 14 to 10.3 ppbv), 32% in Calgary (from 16 to 10.8 ppbv) to 34% in Vancouver (from 14 to 9.2 ppbv), were seen for the grid cells in these cities for which the highest NO_2_ VMR was predicted. Table 3 gives mean, maximum, and minimum percentage changes at the grid-cell level for the COVID-19 scenario for NO_2_, PM_2.5_, and O_3_ concentrations for the grid cells located within each CMA. The average reduction predicted for NO_2_ VMR ranges from 31 to 34% for all four cities in response to the modeled lockdown emission reductions, but it should be noted that these reductions vary across each CMA, from a minimum reduction of 20% up to a maximum reduction of 42%, as a consequence of spatially varying emission changes. Interestingly, the location of the highest percentage change is shifted downwind of the grid cells with the highest NO_*x*_ emissions in each city as a consequence of the complex, nonlinear nature of NO_2_ chemistry (Fig. 5). Note that Griffin et al. (2020) showed similar variations in NO_2_ column values across the Toronto urban area based on an analysis of satellite NO_2_ column measurements.

**Table 3:** Mean, maximum, and minimum percentage changes in mean grid-cell concentrations for the COVID-19 lockdown period for the COVID-19 emission scenario relative to the BAU scenario for the set of grid cells in each metropolitan area

			% Change		
Metropolitan area	# grid cells	Pollutant	Mean	Maximum	Minimum
Montreal CMA	60	NO_2_	−31.2	−41.5	−22.1
		PM_2.5_	−5.6	−18.5	2.6
		O_3_	2.4	11.3	−0.3
Toronto CMA	80	NO_2_	−32.2	−39.0	−24.3
		PM_2.5_	−11.8	−19.6	−6.6
		O_3_	3.0	18.1	−0.3
Calgary CMA	61	NO_2_	−33.7	−41.2	−20.2
		PM_2.5_	−17.4	−22.9	−9.6
		O_3_	3.0	17.3	0.3
Vancouver CMA	40	NO_2_	−30.6	−37.4	−20.3
		PM_2.5_	−14.4	−22.2	−6.9
		O_3_	5.0	21.4	0.6

The maximum predicted PM_2.5_ concentrations, which correspond to grid cells located in the downtown core of each city, have also decreased in the COVID-19 scenario compared to the BAU scenario (Fig. 6). Montreal has the highest PM_2.5_ concentration (13.8 μg m^−3^) among the four cities under the BAU scenario, followed by Vancouver (10.7 μg m^−3^). These peaks can partly be attributed to residential wood combustion, which makes a significant contribution to total PM emissions in these two cities. A less pronounced drop is seen in PM_2.5_ concentrations compared to NO_2_ levels for the COVID-19 scenario. This can be explained by the smaller decrease assumed in this scenario for emissions from the industrial sector (30%), which is the main source of primary PM emissions, compared to the assumed 60% decrease in traffic emissions, which is the dominant source of NO_*x*_ emissions in urban areas (Table 2). As shown in Table 3, the maximum decrease in PM_2.5_ concentration in the four cities for the COVID-19 scenario compared to the BAU scenario ranged from 18 to 22%. The average decrease in PM_2.5_ ranged from 6% for Montreal, 13% for Toronto, 15% for Vancouver, to 17% for Calgary. Interestingly, there is an increase (2–4%) in mean PM_2.5_ concentrations for some Montreal grid cells (e.g., Saint-Jérôme), where residential wood burning is important. This increase in ambient concentration levels reflects the assumed 20% increase in residential heating emissions for the COVID-19 scenario, which in a few locations overwhelmed decreases in primary PM_2.5_ emissions from other source sectors.

Contrary to the decrease in NO_2_ levels during the lockdown, mean O_3_ levels for the COVID-19 scenario were predicted to increase in urban areas and decrease slightly in suburban regions (Fig. 7). A similar response was reported by Menut et al. (2020) for Europe. The maximum percentage increases in O_3_ VMR, which ranged from 11 to 21%, are seen for locations where the maximum decrease in NO_2_ was predicted (Fig. 5). The overall average impact of the lockdown on O_3_ VMR in all four cities is small, however, only 2 to 5% (Table 3). These cold-season increases in mean O_3_ levels are expected in urban areas (i.e., VOC-limited regions) where significant decreases in NO_x_ emissions lead to a reduction in O_3_ titration by NO. However, there were a few grid cells in Montreal and Toronto near the CMA boundaries where mean O_3_ levels were predicted to decrease for the COVID-19 scenario (Fig. 7). These decreases may be linked to reduced downwind O_3_ production in the urban plume and the non-linear response of ozone to NO_*x*_ emission reduction depending on the ratio of VOC/NO_*x*_ (e.g., Sillman 1999; Sillman et al. 2003).

## Discussion

Surface NO_2_, PM_2.5_, and O_3_ measurements for the four CMAs for the pre-lockdown and lockdown periods in 2020 are compared in Figs. 2 and 3 with 2010–2019 measurements for the same periods. Some pronounced differences were evident for 2020; however, it was not clear to what extent COVID-19-related emission changes contributed to these differences compared to meteorological variations. Since the two modeled emission scenarios discussed in the “Modeling results” section used the same 2020 meteorology and thus isolate the impacts of COVID-19-related emission changes, it is of interest to compare model predictions of AQ impacts from the two scenarios against the estimates from surface measurements.

Figure S7 is similar in form to Fig. 2, but it compares city-wide-average time series of 2020 surface observations of NO_2_, PM_2.5_, and O_3_ with model predictions from the two scenarios for the pre-lockdown, transition, and lockdown periods for each of the four CMAs. The comparison of model predictions from the BAU scenario with measurements for the pre-lockdown period (i.e., up to 14 March) gives an indication of pre-lockdown model skill in these four urban areas. Model NO_2_ VMR predictions for the pre-lockdown period are generally good for Montreal, Toronto, and Vancouver but are biased high for Calgary. This performance is consistent with the NO_2_ scores for winter 2020 for both western and eastern Canada shown in Table S2. Model PM_2.5_ concentration predictions are very good for Toronto for the pre-lockdown period but are biased high for the other three cities; Table S2 lists small overall biases for PM_2.5_ for winter 2020 when observations from all PM_2.5_ measurement stations in western Canada and in eastern Canada were considered (−0.6 and 0.3 μg m^−3^, respectively). Lastly, model O_3_ predictions are generally good for Toronto and Vancouver for the pre-lockdown period but are biased low for Montreal and Calgary. Table S2 has a negative bias for O_3_ in eastern Canada (−2.1 ppbv), consistent with the pre-lockdown O_3_ time series for Montreal and Toronto in Figure S7, and a small positive bias for western Canada (0.3 ppbv), consistent with the pre-lockdown O_3_ time series for Vancouver but not Calgary in Figure S7.

As expected from Figs. 5–7 and Table 3, the BAU time series for NO_2_ and PM_2.5_ are consistently higher than the corresponding COVID-19 time series for the four CMAs while the BAU time series for O_3_ are consistently lower than the COVID-19 time series. Overall, the COVID-19 scenario results for NO_2_ show better agreement with the observations than the BAU results for Montreal and Calgary; for PM_2.5_, the same is true for Montreal, Toronto, and Vancouver, and for O_3_, it is true for all four cities. This suggests that the COVID-19 scenario emissions better represent actual emissions during the lockdown period.

Figure S8 is similar in form to Fig. 3, but it compares mean diurnal time series of 2020 surface observations of NO_2_, PM_2.5_, and O_3_ for the lockdown period with model predictions from the two emission scenarios for each of the four CMAs. Again, the COVID-19 scenario predictions for NO_2_ are in better agreement with the observations than the BAU results for Montreal and Calgary; for PM_2.5_, the same is true for Montreal, Toronto, and Vancouver, and for O_3_, it is true for all four cities.

Table S3 compares mean hourly NO_2_, PM_2.5_, and O_3_ surface concentrations for the lockdown period averaged over all measurement stations in each CMA with model-predicted values at the same locations for the two emission scenarios. Seventy-five percent of the COVID-19 scenario values were closer to the measured values than the BAU scenario values. This table also presents the predicted average impact of the lockdown on pollutant levels in each CMA at AQ measurement station locations. Surface concentrations for the COVID-19 scenario were lower by 30–38% for NO_2_ and by 15–21% for PM_2.5_, with the largest changes in Vancouver and Calgary, respectively. O_3_ levels were higher by 6–10%, with the largest changes in Calgary. Note that the predicted decreases for NO_2_ are considerably larger than the range of 6–17% suggested by the analysis of surface AQ measurements (Table 1). The same is true for PM_2.5_, for which the analysis of surface AQ measurements suggested a decrease of 4% for Montreal but an increase for the other three cities relative to previous years. These differences point to the confounding impact of meteorological patterns and conditions, which masked the effect of the lockdown measures based on a direct analysis of AQ surface measurements. Goldberg et al. (2020) came to a similar conclusion in an analysis of satellite NO_2_ column measurements over North America for spring 2020.

**Fig. 8:**
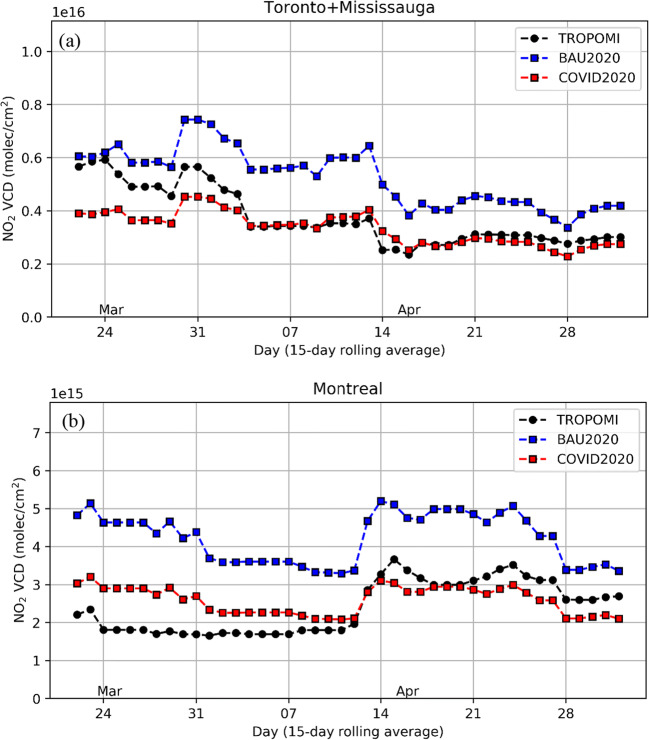
15-day running mean of NO_2_ vertical column density for the 22 March–2 May period over (**a**) Toronto-Mississauga and (**b**) Montreal comparing the TROPOMI observations (black), with the model BAU (blue) and COVID-19 lockdown scenario (red) values

Satellite observations were discussed in the “Introduction” section as another valuable source of air quality measurements. Model predictions from the BAU and COVID-19 emission scenarios have also been compared to NO_2_ vertical column density (VCD) observations made by the Tropospheric Ozone Monitoring Instrument (TROPOMI). Figure 8 compares a time series of 15-day running mean observed TROPOMI daily NO_2_ column density values in Toronto and Montreal during the lockdown period with model NO_2_ columns sampled at each TROPOMI pixel at the satellite overpass time and transformed with the satellite retrieval averaging kernel. More details about this comparison can be found in Griffin et al. (2020). It is evident that the BAU model run overestimates column NO_2_ for the entire lockdown period in both cities, whereas the COVID-19 scenario with reduced emission run shows much better agreement with the observed TROPOMI values for both cities. Griffin et al. (2020) estimated that lockdown measures reduced the average NO_2_ column over Toronto by about 40%, while Goldberg et al. (2020), in a closely related study, reported a 42% reduction in the average NO_2_ column over Toronto and a 30% reduction for Montreal. These results are similar to the model-predicted reductions of 32% and 31% in average surface NO_2_ in Table 3. As well, Figures S10 and S11 compare mean NO_2_ column fields observed by TROPOMI with the model-predicted mean NO_2_ column fields for the COVID emission scenario for the Montreal and Toronto regions for the latter part of the lockdown period. The qualitative agreement of the spatial patterns from the satellite and the model is very good.

Photochemistry is a complicating factor for interpreting NO_2_ and O_3_ lockdown responses from both surface measurements and model predictions for the two emission scenarios. For example, NO_2_ levels are consistently lower across each urban area for the COVID-19 vs. the BAU scenario, but grid-cell O_3_ differences range from a decrease of 0.3% to an increase of 21% (Table 3). The spatial distributions of relative differences between the two emission scenarios display even more variation: relative differences of mean NO_2_ VMR are all positive in Fig. 5, indicating higher NO_2_ levels for the BAU scenario, but relative differences of mean O_3_ levels are positive within the CMA boundaries though largely negative in the surrounding areas (Fig. 7). We know that NO_2_ and O_3_ are tightly coupled through the photostationary state in the daytime and also at night via NO titration (e.g., Brown et al. 2006; Clapp and Jenkin 2001). For this reason, we also looked at odd oxygen (O_*x*_), which we defined as O_3_+NO_2_ (neglecting several nocturnal species: see Brown et al. 2006). This quantity varies less than either NO_2_ or O_3_ individually and is quasi-conserved for unchanging emissions (e.g., Lee et al. 2020). As shown in Figures S7d and S8d, a slight decrease in mean O_*x*_ levels is seen for the COVID-19 scenario in all four cities, indicating that the net impact of reduced NO_*x*_ emissions during the lockdown period is a decrease in O_*x*_ levels. Figure S9 presents the mean O_*x*_ fields for the two emission scenarios plus their actual difference and relative difference fields. The O_*x*_ concentration relative difference fields in Figure S9 are more like the NO_2_ relative difference fields in Fig. 5 than the O_3_ difference fields in Fig. 7 in that they are also consistently positive. One key difference, though, is that the largest O_*x*_ relative differences occur over the urban cores where we expect the largest changes in NO_*x*_ and primary PM_2.5_ emissions to occur. Consequently, the O_*x*_ concentration relative difference fields for the four CMAs are more similar to the PM_2.5_ relative difference fields (Fig. 6) than they are to the NO_2_ relative difference fields (Fig. 5).

The comparisons of GEM-MACH model predictions for the two emission scenarios with surface and satellite air quality measurements presented in Figs. 5–8 and S7–S8 and in Table S3 suggest that the COVID-19 emission scenario agrees better overall with measurements for the lockdown period in Canada than the counterfactual BAU emission scenario. These results suggest in turn that the assumptions made regarding sectoral emission changes for the COVID-19 scenario, including reductions in traffic emissions, may be reasonable (see also Figures S10 and S11). There are, however, many uncertainties associated with the COVID-19 scenario emissions. The assumed reductions in aviation emissions may have the least uncertainty since the key activity associated with this sector, the number of daily aircraft landings and takeoffs, is known with high certainty for each large airport (Statistics Canada 2020). Traffic-activity data (Apple Inc, 2020) and traffic-count data from various municipalities also support the estimates of overall emission reductions from traffic, although there is greater uncertainty about disaggregated activity levels by vehicle class and road type, particularly outside the cities. Changes in industrial and residential emissions are more uncertain, and emissions from other source types, which are significant (Table 2), were simply assumed in this study not to change due to COVID-19 lockdown measures. While the results presented here are promising, they are still preliminary and they cannot confirm that the assumed sector-specific emission reductions are correct. The development of a more comprehensive and realistic lockdown emission scenario, however, will have to wait for the development and release of a Canadian APEI for the 2020 data year (expected sometime in 2022).

Another source of uncertainty is the treatment of the US emissions, which were assumed to remain unchanged for the COVID-19 scenario due to the challenge and complexity of representing the initial US response in spring 2020 to the COVID-19 pandemic. Other studies, though, have shown improvements in air quality in many US cities during this period (e.g., Berman and Ebisu 2020; Goldberg et al. 2020; Schindler 2020). US emissions do have an impact on Canadian air quality (e.g., Olson et al. 1992; Roelofs 1993; Yap et al. 1988). Further simulations are needed to account for emission changes in the USA as well as in Canada due to lockdown measures in spring 2020. However, the potential impact of US emissions on the four Canadian cities considered in this study varies greatly. Due to topography, Vancouver is located in an isolated airshed not unlike Los Angeles. Calgary and Montreal are both located some distance from the US border (Fig. 4), and emissions in northwestern USA are relatively low (U.S. EPA 2014), further reducing the potential impact of US emissions on Vancouver and Calgary. Thus, Toronto is the most likely of the four to be impacted by US emissions given its proximity to US states on three sides and to the industrialized Ohio Valley, so particular attention will need to be paid to Toronto and the rest of southern Ontario in any future COVID-19 simulations that consider US emission changes.

## Summary and conclusions

In the first part of this study, the impact of COVID-19 on air quality in Canada during the lockdown period in spring 2020 was investigated using an analysis of ground-level measurements in four major Canadian cities: Montreal, Toronto, Calgary, and Vancouver. Declines in surface NO_2_ levels between the pre-lockdown and lockdown periods for these cities were 6 to 17% greater than those observed in the preceding decade (2010–2019). However, the impact of the COVID-19 lockdown on measured PM_2.5_ and O_3_ surface concentrations was less pronounced. It was challenging to quantify the pollution decreases due to COVID-19 lockdown measures using only observations due to the difficulty of filtering out the impact of seasonal and interannual variations in meteorology on the measured concentrations (e.g., Goldberg et al. 2020).

To avoid the confounding influence of meteorology, an emission scenario analysis was performed with the GEM-MACH chemical transport model to quantify the impact of reduced emissions due to the lockdown on NO_2_, PM_2.5_, and ozone surface levels. Two emission scenarios were considered, a “business-as-usual” scenario that accounted for expected seasonal variations in emissions but not the impact of the lockdown, and a COVID-19 emission scenario that included estimated emission changes, both decreases and increases, due to the lockdown across Canada for four emission source sectors. Emissions from the industrial sector were assumed to decrease by 30% based on a 50–60% decrease in workplace activity (Figure S2); emissions from traffic were reduced by 60% based on aggregated smartphone mobility data and traffic-count data for the lockdown period (Figure S1); aviation emissions were reduced by 80% based on government landing and takeoff statistics; and residential heating emissions were increased by 20% based on smartphone mobility data that showed the population in each province spending 20% more time at home during the lockdown (Figure S2). Changes in US emissions due to lockdown measures were not considered in this study. The GEM-MACH model was run on a North American grid with 10-km grid spacing from 1 February to 11 May 2020 for the BAU scenario and from 15 March to 11 May 2020 for the COVID-19 scenario.

By comparing model predictions for the two emission scenarios, surface NO_2_ levels in the four major urban areas were found to have decreased by 31 to 34% on average for the COVID-19 scenario, but with spatial variations across each city that ranged from a minimum decrease of 20% to a maximum decrease of 42% (Table 3). PM_2.5_ concentrations also decreased, but to a lesser extent, from 6 to 17% on average for the four cities, and ranging from an increase of 3% to a maximum decrease of 23%. This difference is due to the fact that traffic emissions, which were assumed to decrease by 60%, make a smaller contribution to overall PM_2.5_ emissions than they do to NO_2_ emissions. Also, the PM_2.5_ emissions for other sectors, such as industry and residential heating, had smaller decreases or even increased (Table 2). By contrast, O_3_ levels for the four cities increased by 2 to 5% on average, with a maximum increase of 21% in the urban cores and a maximum decrease of 0.3% in the suburbs (Table 3). To remove the complication of photochemistry, odd oxygen (O_*x*_), which was defined as the sum of O_3_ and NO_2_, was also examined. Interestingly, the spatial distribution of the O_*x*_ decreases resembled the spatial distribution of PM_2.5_ decreases more than it did those for its two constituents, O_3_ and NO_2_.

GEM-MACH model predictions for the two emission scenarios were also compared to surface and satellite measurements. Overall, the model predictions for the COVID-19 scenario agreed better with measurements than those for the BAU scenario, suggesting that the emission reductions assumed for the COVID-19 scenario better represented the impact of the lockdown period on emissions across Canada than the counterfactual BAU scenario, which served as a sort of null hypothesis. The AQ impacts due to the lockdown that were estimated by the model emission scenarios were also larger than those estimated directly from surface measurements. These findings point to the confounding influence of meteorological variations on attempts to isolate the impact of lockdown-related emission changes. They are consistent with the argument of Goldberg et al. (2020) that meteorological conditions in spring 2020 over North America were more favorable for better air quality than the meteorological conditions in 2019, including reduced snow cover extent and snow depth.

## Supplementary Information

ESM 1 (DOCX 12852 kb)
