# Supplementary material for: Isolating the impact of COVID-19 lockdown measures on urban air quality in Canada
Source: Air Qual Atmos Health. 2021 May 18;14(10):1549–70. doi: 10.1007/s11869-021-01039-1 (PMC8130219; doi:10.1007/s11869-021-01039-1)
Supplement: Supplementary file 1 — (DOCX 12852 kb) [file 11869_2021_1039_MOESM1_ESM.docx]

Supplement to:

**Isolating the impact of COVID-19 lockdown measures on urban air quality in Canada**

Rabab Mashayekhi^1*^, Radenko Pavlovic^1^, Jacinthe Racine^1^, Michael D. Moran^2^, Patrick M. Manseau^1^, Annie Duhamel^1^, Ali Katal^1^, Jessica Miville^1^, David Niemi^1^, Si Jun Peng^1^, Mourad Sassi^1^, Debora Griffin^2^ and Chris McLinden^2^

^1^Air Quality Policy-Issue Response Section, Canadian Meteorological Center, Environment and Climate Change Canada, Dorval, Quebec, Canada

^2^Air Quality Research Division, Environment and Climate Change Canada, Toronto, Ontario, Canada

*Corresponding author. rabab.mashayekhi@canada.ca

19 March 2021

**S1. Quality assurance/quality control of near-real-time hourly air quality measurements**

The quality assurance/quality control (QA/QC) program for near-real-time hourly air quality concentration measurements ingested by the ECCC Automatic Data Extraction (ADE) system determines whether hourly observations are classified as ‘Good’, ‘Suspect’, or ‘Bad’. Only the observations classified as “Good” were kept for the analysis in this paper. The QA is done by running two tests: (1) a threshold test; and (2) a jump test.

*Threshold Test*

The threshold test checks whether observations fall outside of a range pre-specified for each species. All values are submitted to this test regardless of their status. Two sets of concentration thresholds are considered, a minimum-maximum range to distinguish between good and suspect values and a second, wider minimum-maximum range to distinguish between suspect and bad values. The concentration thresholds used for O_3_, NO_2_, and PM_2.5_ are provided in Table S1.

Because slightly negative concentration values can be reported at very low concentrations from improperly calibrated devices, both the ‘Suspect Minimum’ and ‘Error Minimum’ thresholds are set to small negative values. As a consequence, negative concentration values can be flagged as ‘Good’ if they are larger than the ‘Suspect Minimum’ threshold or ‘Bad’ if they are smaller (i.e., more negative) than the ‘Error Minimum’ thresholds. Note that no ‘Suspect’ values exist below 0 because the ‘Suspect Minimum’ and ‘Error Minimum’ thresholds are set to the same small negative values.

Table S1. Species-specific values used by ADE system for threshold test and jump test.

|  |  | **Threshold Test Values** | | | | **Jump Test Values** | |
| --- | --- | --- | --- | --- | --- | --- | --- |
| **Species** | **Units** | **Error**  **Minimum** | **Suspect**  **Minimum** | **Suspect**  **Maximum** | **Error**  **Maximum** | **Suspect**  **Threshold** | **Maximum Threshold** |
| O_3_ | ppbv | -3.0 | -3.0 | 300 | 500 | 60 | 200 |
| NO_2_ | ppbv | -3.0 | -3.0 | 200 | 2000 | 30 | 100 |
| PM_2.5_ | µg m^-3^ | -3.4 | -3.4 | 300 | 1000 | 90 | 200 |

*Jump Test*

The jump test is used to determine if there is an abrupt change (jump) between the current and the previous observation. Only the observation from the previous hour is used for the comparison and is obtained directly from the database.

Only ‘Good’ observations are submitted to this test. If both the current and the previous observations are ‘Good’, then the difference between the two is calculated. If the difference is greater than the ‘Suspect Threshold’, then the QC flag is set to ‘Suspect’. However, if the previous observation is not ‘Good’ or is not available, then the QC flag is set to ‘Suspect’ if the current observation is above the ‘Maximum Threshold’ limit specified for that species.

Note that this jump test will only catch isolated peak values or “spikes”. Additional checks would be needed if a bad sequence of data were sent, including an extended sequence of identical values.

**S2. Impact of COVID-19 measures on societal activity in Canada in spring 2020**

A number of web applications are available that provide traffic and location data based on anonymized aggregation of signals from individual smartphones being carried by individuals in moving vehicles or in buildings or other places. Three large companies (Apple, Google, and Waze) have made global mobility data sets available for 2020 for the pre-lockdown, lowdown, and post-lockdown periods. Figures S1, S2, and S3 show different pre-lockdown and lockdown mobility analyses for the four Canadian cities considered in this paper or for the four provinces in which they are located. Aggregated city-level traffic activity time series are presented in Figures S1 and S3; each figure shows similar temporal changes between the cities and the two figures show broadly similar behavior despite being based on different smartphone samples.

Figure S4 shows a comparison of time series of two-way daily traffic counts for the same period in 2019 and 2020 at one location near downtown Calgary. The values were obtained by an automatic traffic recorder so all vehicles were considered, not just those vehicles carrying smartphones with particular apps installed. This analysis is also for a single location, not for an entire city, and it compares traffic between two years, not for two periods within the same year. However, this figure, like the first three, shows a similar large decrease in traffic for the 2020 lockdown period. Other traffic-count data sets were also obtained for this study from agencies in Montreal, Toronto, and Vancouver, but as these data are not accessible publicly, we have chosen not to show comparable analyses to Figure S4.

1.
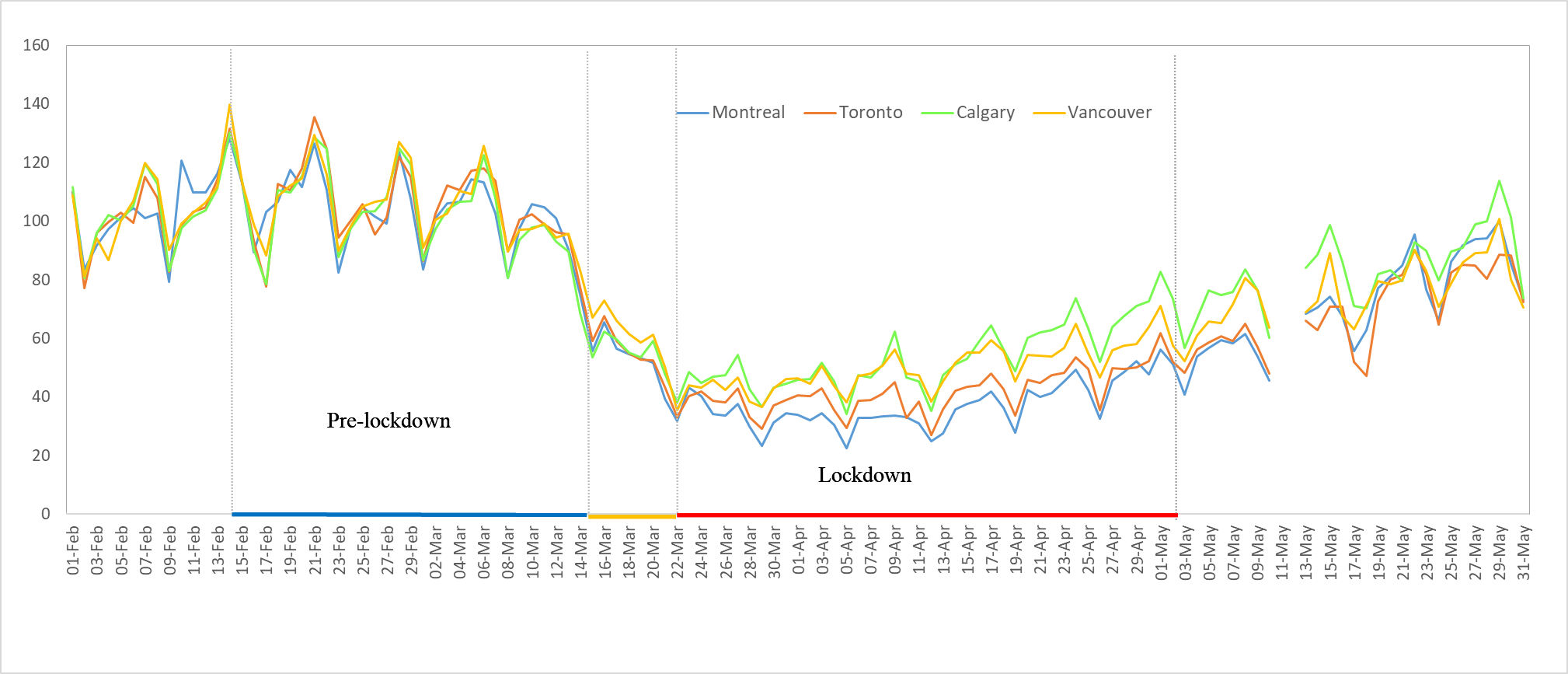
Driving


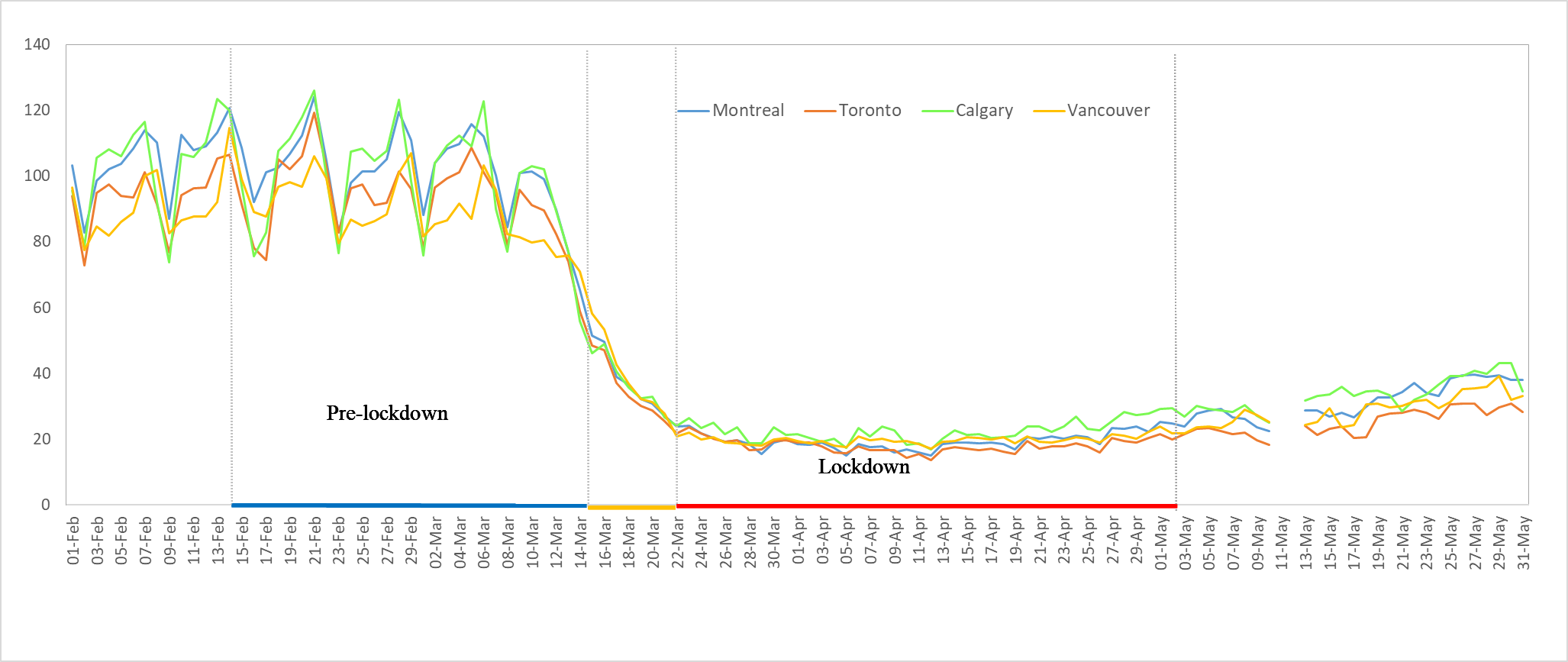
(b) Transit

Figure S1. Time series of normalized daily traffic data extracted from Apple Mobility Trends Reports (Apple Inc., 2020) for (a) driving and (b) transit activities for the four-month period 1 February‒31 May 2020 for Montreal, Toronto, Calgary, and Vancouver. The plotted values are relative to the number of user requests for 13 January 2020, for which have been normalized to 100.

Figure S2. Percentage change in visitors to (or time spent in) categorized community places by province during the lockdown period (22 March‒2 May 2020) compared to median visitors to (or time spent in) the same categorized places for a pre-lockdown period (3 Jan. ‒6 Feb. 2020) for six different activity categories. The four provinces shown include Montreal (Quebec), Toronto (Ontario), Calgary (Alberta), and Vancouver (British Columbia), respectively. The data shown are based on Google Community Mobility Reports (Google, 2020).


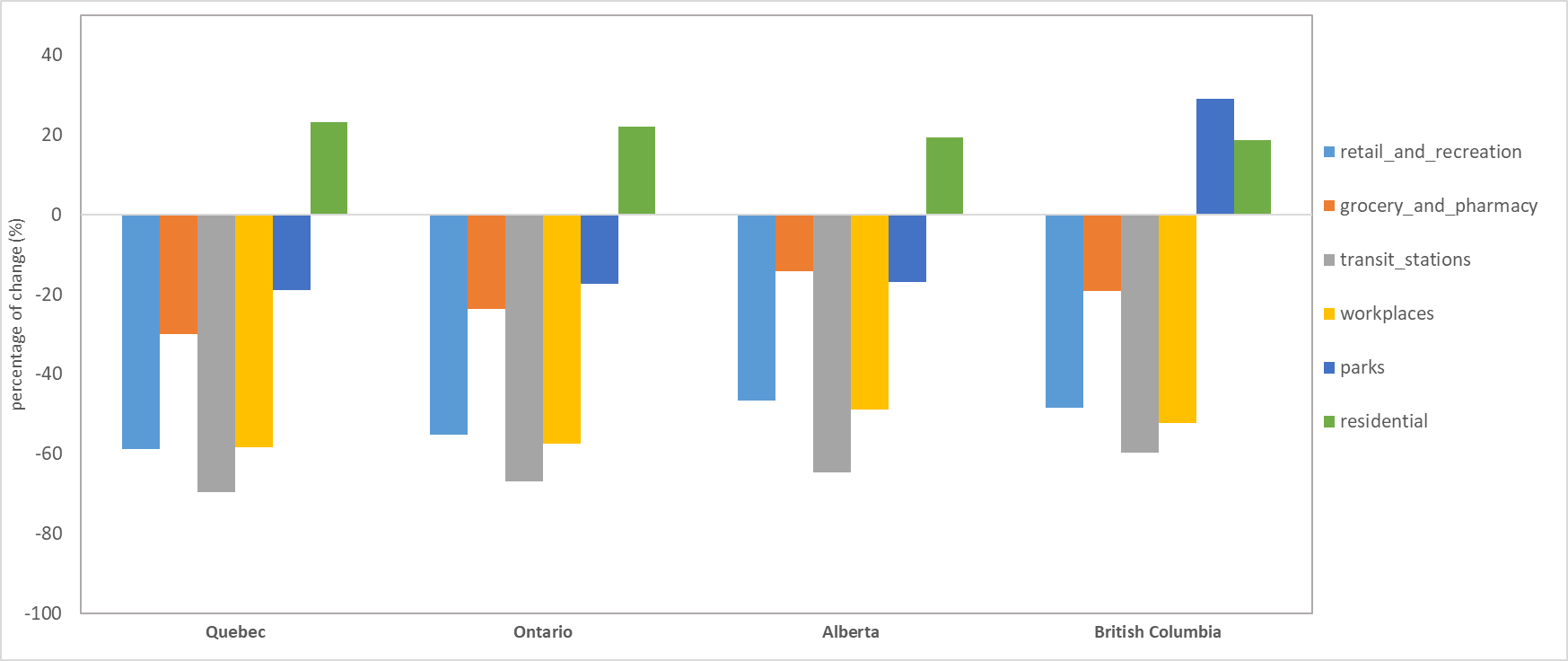


Quebec

Ontario

Alberta

British Columbia


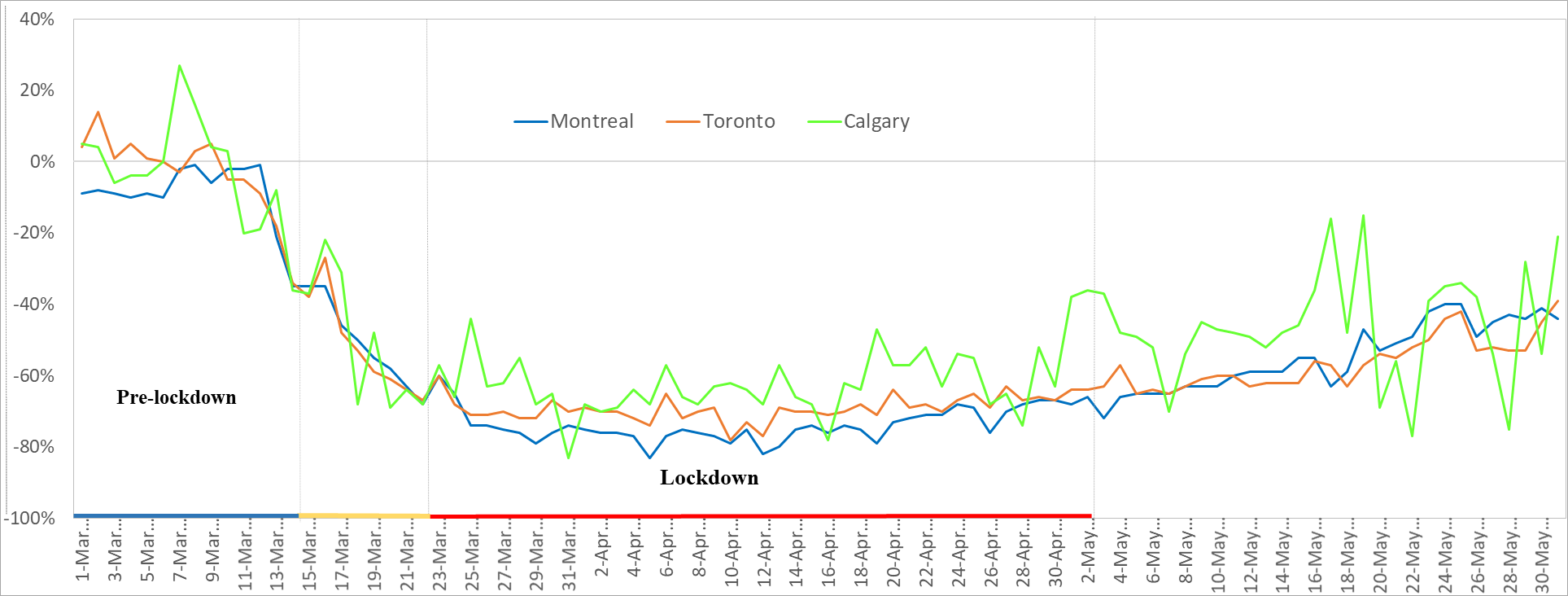
Figure S3. Time series of normalized daily traffic data extracted from Waze COVID-19 local driving trends (Waze Mobile., 2021; <https://www.waze.com/covid19>) for the three-month period from 1 March to 31 May 2020 for Montreal, Toronto, and Calgary. The baseline is the average value for the corresponding day of the week during the 2- week period 11‒25 February 2020. Note that the data for Vancouver were not available.


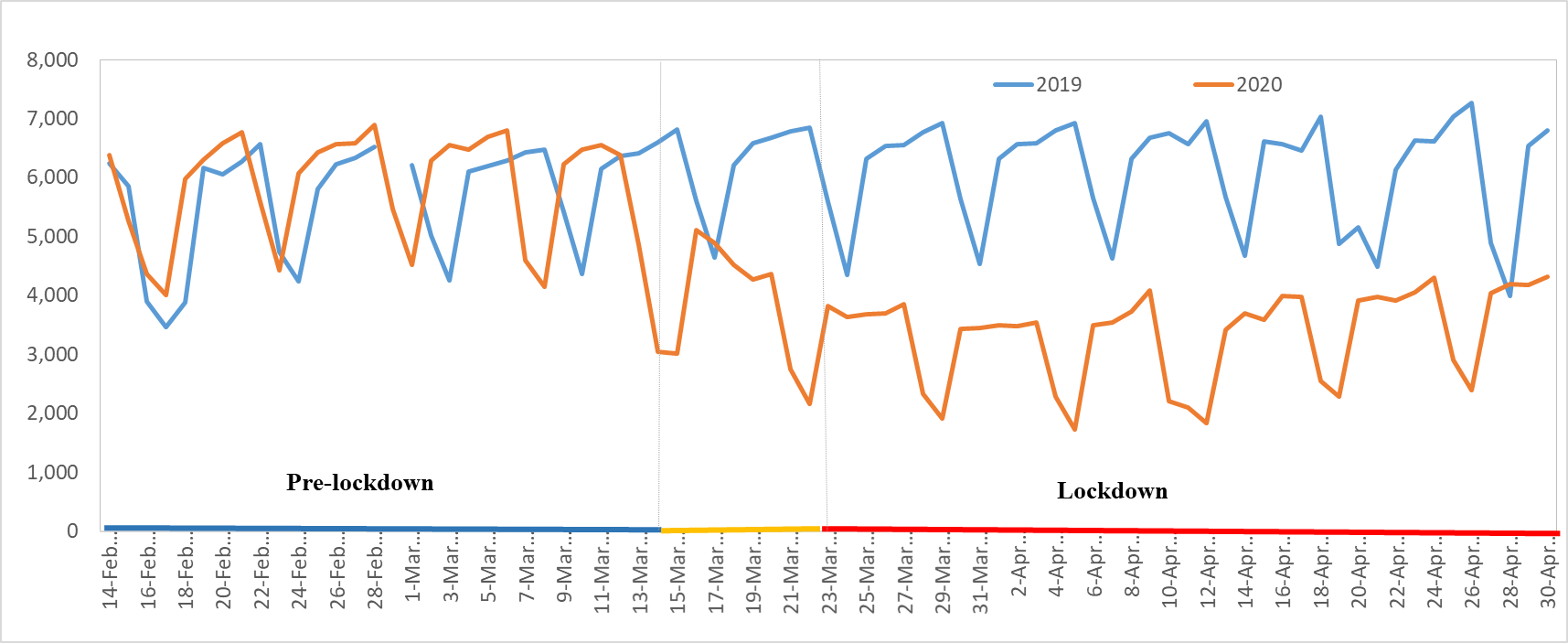


Figure S4. Comparison of time series of daily average number of cars (two ways) at a location near downtown Calgary (automatic traffic reader #60021520) for a period from 14 February to 30 April in 2019 versus 2020. The raw traffic count data were accessed from a Province of Alberta public website (<http://www.transportation.alberta.ca/mapping/>).

**S3. Relevant climatological factors in spring 2020 in Canada**


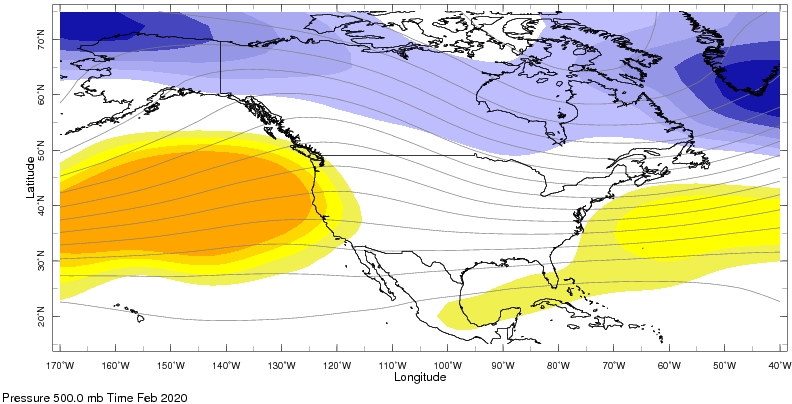

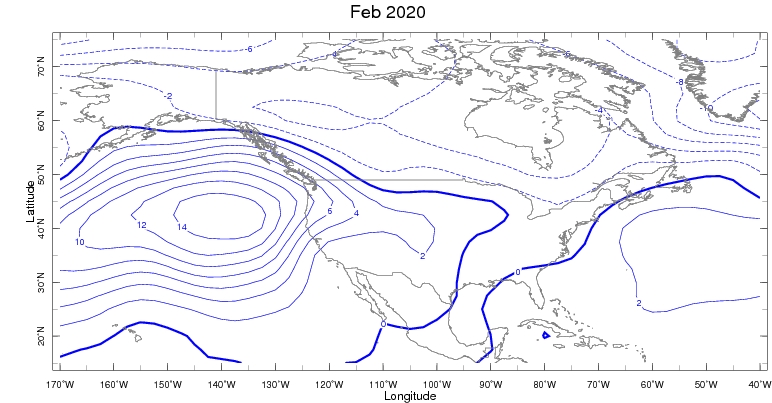

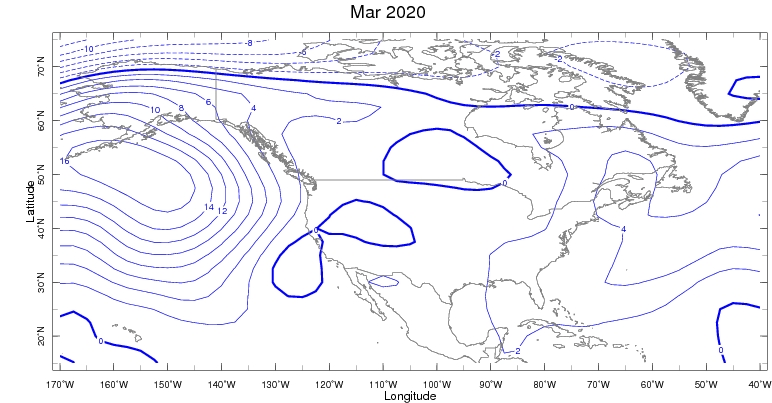

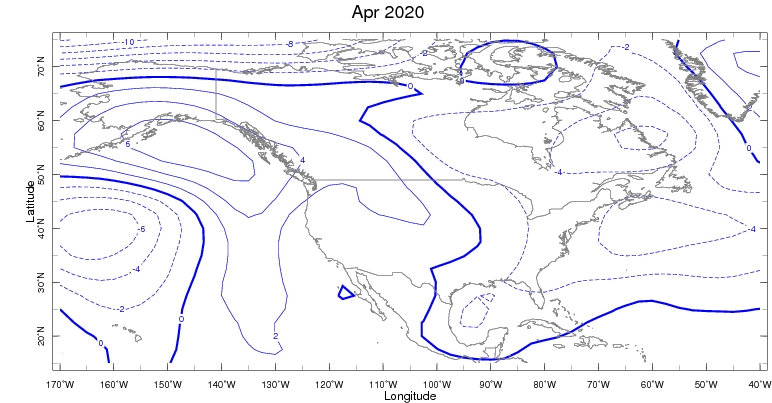

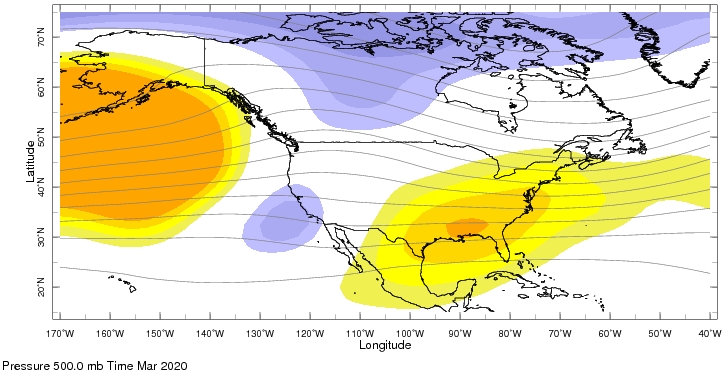

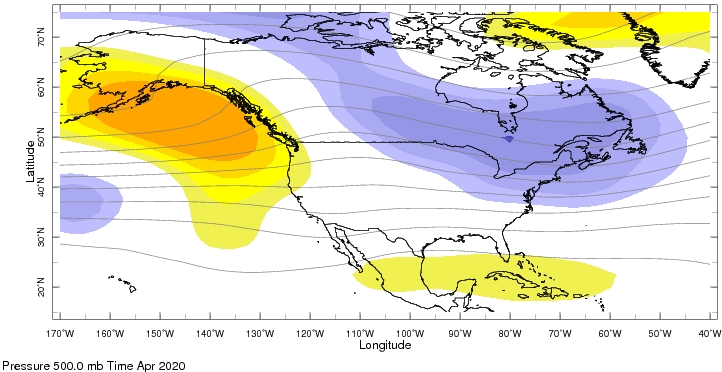


Feb 2020

March 2020

April 2020

1. Sea level pressure

(b) 500 hPa geopotential height

Figure S5. North American monthly average (a) sea level pressure and (b) 500 hPa geopotential height anomalies with respect to 1981-2010 climatology for March, April, and May 2020 (data source: IRI, 2020).

**S4. GEM-MACH performance evaluation results for recent time periods**

Table S2. Hourly performance statistics for GEM-MACHv3.0 72-hour forecasts of NO_2_, O_3_, and PM_2.5_ for winter 2020 (15 Dec. – 28 Feb.) and summer 2019 (15 June–31 Aug.) for western Canada (WCAN) and eastern Canada (ECAN). Three statistics are presented: mean bias (MB); Pearson correlation coefficient (R); and root mean square error (RMSE).

|  | **Hourly** | **Winter 2020** | | **Summer 2019** | |
| --- | --- | --- | --- | --- | --- |
| **Pollutant (Unit)** | **Statistic** | **WCAN** | **ECAN** | **WCAN** | **ECAN** |
|  | MB | -0.8 | -0.5 | 0.1 | 0.2 |
| NO_2_ (ppbv) | R | 0.61 | 0.67 | 0.51 | 0.57 |
|  | RMSE | 8.5 | 6.6 | 4.3 | 4.6 |
|  | MB | 0.3 | -2.1 | -5.7 | -3.9 |
| O_3_ (ppbv) | R | 0.61 | 0.65 | 0.67 | 0.72 |
|  | RMSE | 10.4 | 8.1 | 10.2 | 9.9 |
|  | MB | -0.6 | 0.3 | -2.5 | -2.8 |
| PM_2.5_  (ug m^-3^) | R | 0.26 | 0.54 | 0.1 | 0.22 |
|  | RMSE | 8.5 | 9.6 | 6.3 | 7.1 |

**S5. Additional analyses of model predictions for pre-lockdown and lockdown periods in spring 2020**

Figure S6 shows mean difference fields for NO_2_, O_3_, and PM_2.5_ surface volume mixing ratio (VMR) based on model predictions for the COVID-19 emissions scenario and for a second COVID-19 emissions scenario that used the same scaled Canadian emissions but different U.S. emissions. The latter were obtained from a 2028 projected National Emissions Inventory (NEI) that was based on version 1 of the 2016 U.S. NEI (see <https://www.epa.gov/air-emissions-modeling/2016v1-platform>). U.S. emissions are projected to decline overall between 2017 and 2018, so all of the panels in Figure S6 show declines in pollutant concentrations, with the exception of O_3_ in urban areas, where lower NO_x_ emissions have reduced NO titration and increased O_3_ VMR levels. This figure gives some sense of the impact that reduced emissions in the U.S. due to COVID-19 measures might have had in the U.S. and in the southern border regions of Canada.

Figures S7, S8, and S9 show different analyses comparing model predictions for the two emission scenarios with observations for each of Montreal, Toronto, Calgary, and Vancouver. Figure S7 shows measured and modelled time series of rolling 7-day concentration averages for the pre-lockdown and lockdown periods and is comparable to Figure 2. Figure S8 shows measured and modelled diurnal time series for the full lockdown period and is comparable to Figure 3. NO_2_, PM_2.5_, and O_3_ are again considered in both Figures S7 and S8, but an additional quantity, odd oxygen (O_x_ =O_3_+NO_2_), has now been added. Figure S9 shows spatial distributions of mean hourly O_x_ for the full lockdown period and is comparable to the sum of Figures 5 and 7.

Figures S10 and S11 compare observed mean TROPOMI NO_2_ vertical column density fields for part of the lockdown period for the Montreal and Toronto regions, respectively, with equivalent model-predicted mean NO_2_ VCD fields at the satellite overpass time for the same period that were obtained from predicted NO_2_ VMR fields.  The methodology described in Griffin et al. (2020) was used to calculate the model-predicted NO_2_ VCD fields.  The qualitative agreement between the observed and predicted mean NO_2_ VCD fields is very good.  Since NO_2_ is a primary pollutant, this suggests that the emission reductions used for the COVID emissions scenario are reasonable, including sectoral variations since different emissions sectors have different spatial patterns but the overall spatial patterns predicted by the model for this scenario are similar to the observed spatial patterns.


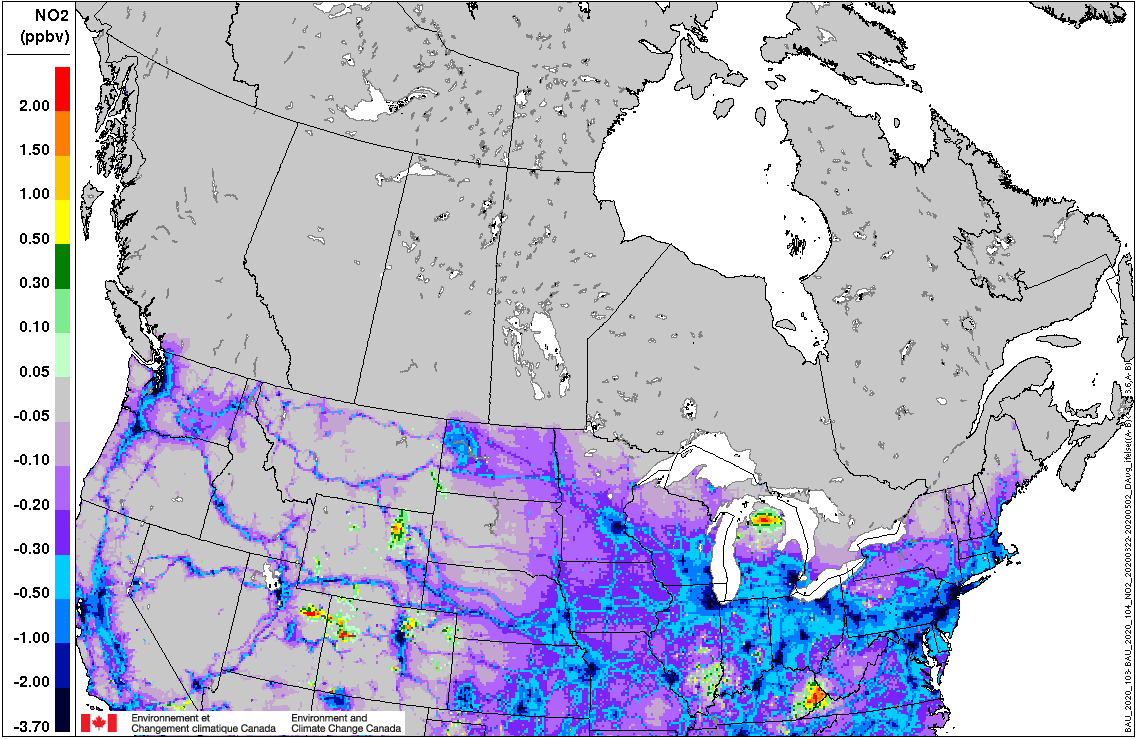

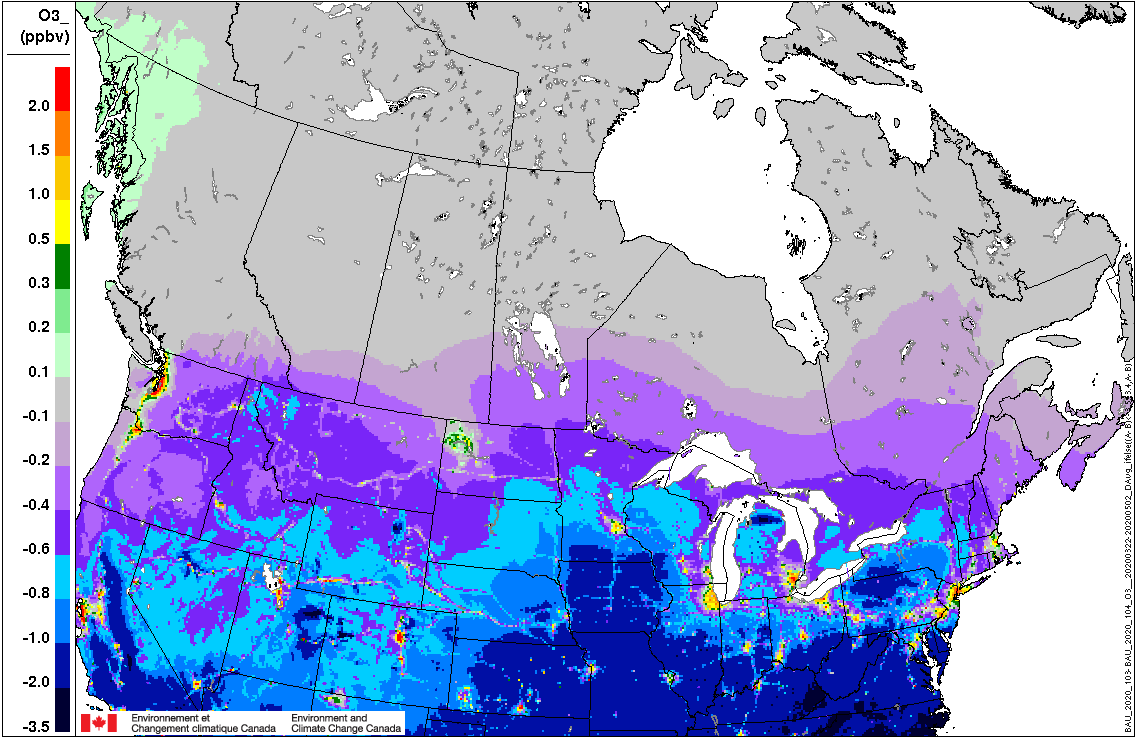

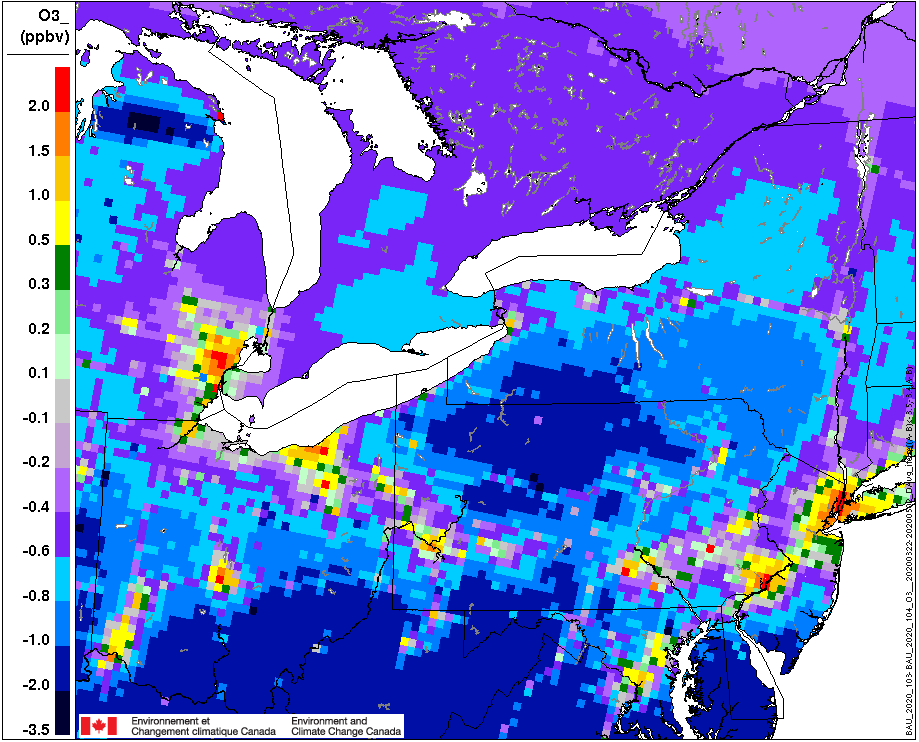

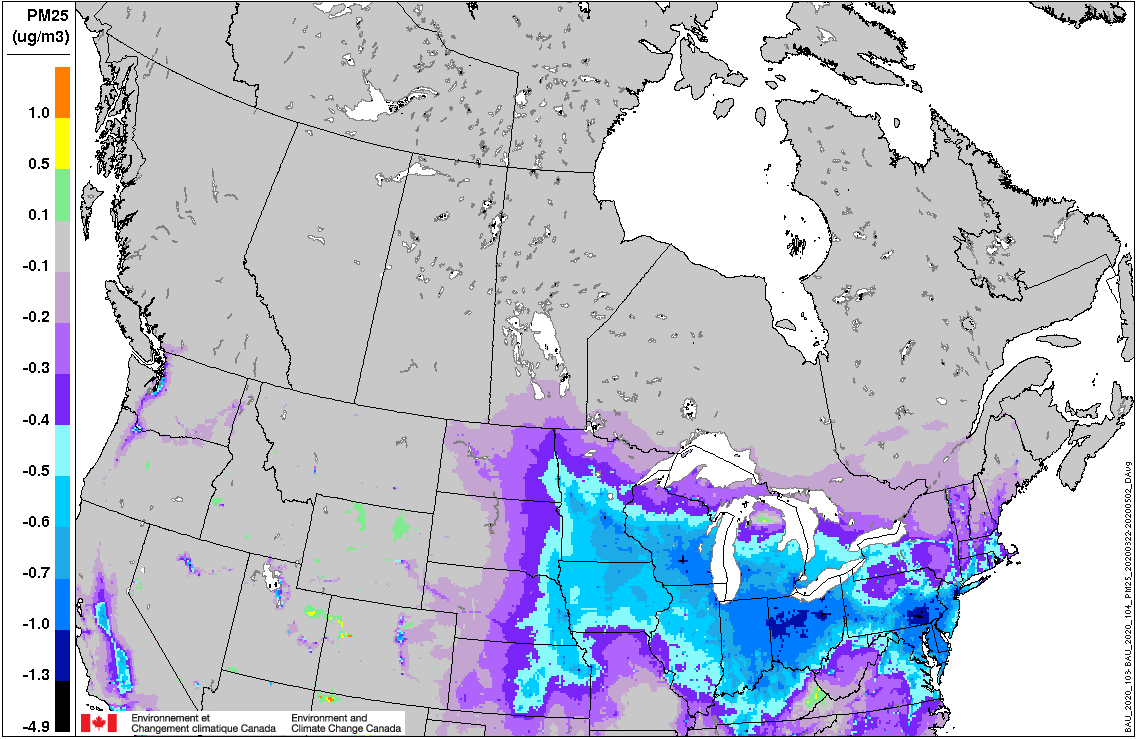

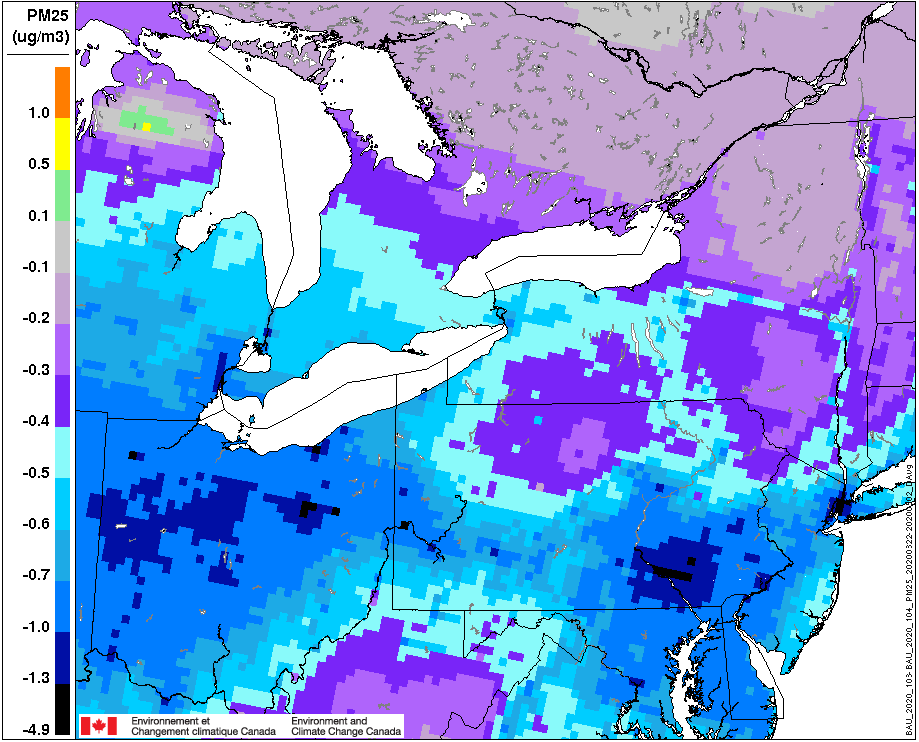

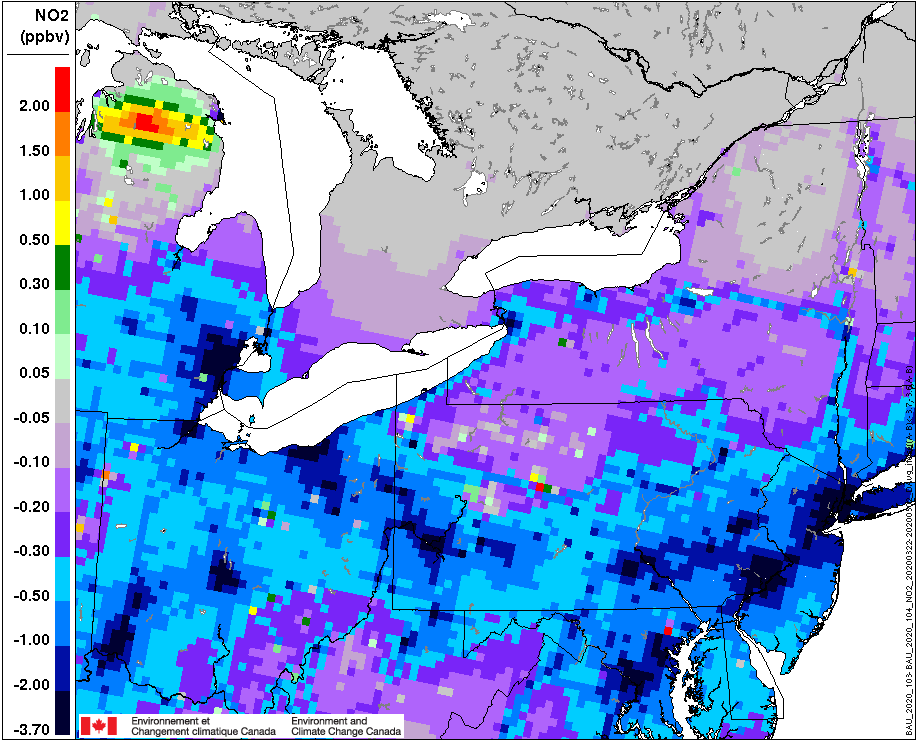


1. NO_2_ (ppbv)
2. O_3_ (ppbv)

(c) PM_2.5_ (µg m^-3^)

Figure S6. Mean hourly (a) NO_2_, (b) O_3_, and (c) PM_2.5_ surface VMR and concentration difference fields for the ‘full lockdown’ period (22 March‒2 May 2020) for model prediction that used two different U.S. emissions inventories (run with projected 2028 emissions minus run with projected 2017 emissions). The left panels show the spatial difference over Canada and U.S., while the right panel is zoomed over southern Ontario.

1. NO_2_ (ppbv)


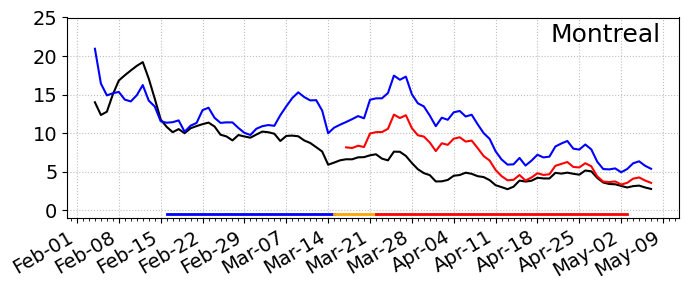

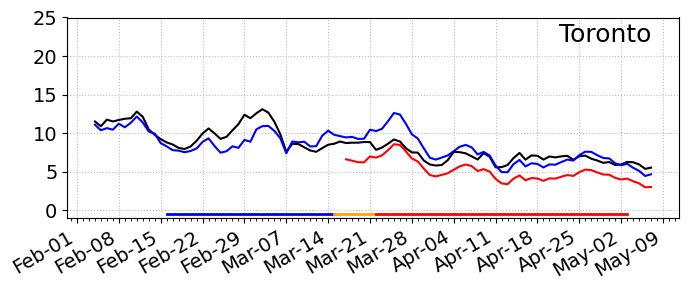

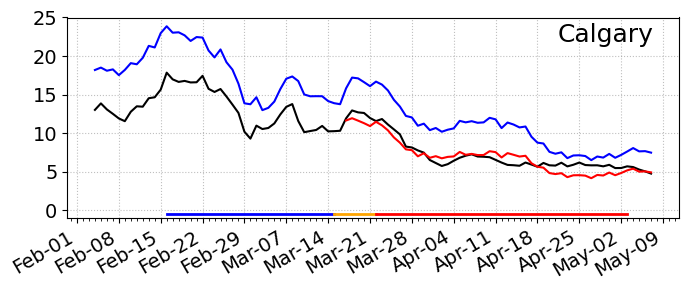

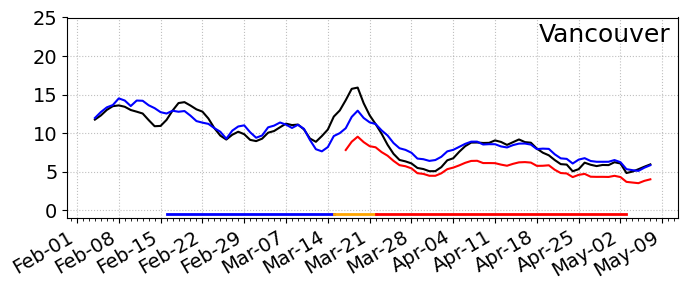


1. PM_2.5_ (µg m^-3^)


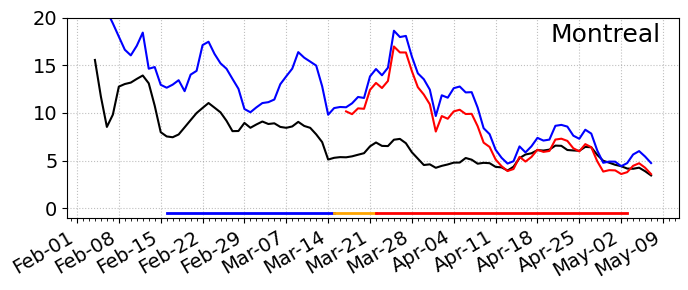

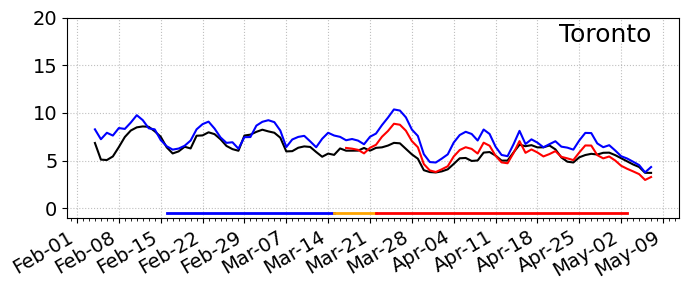

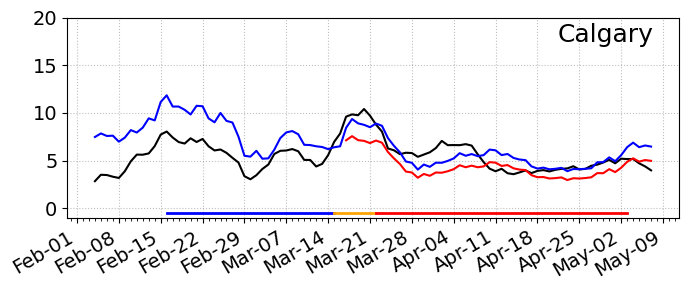

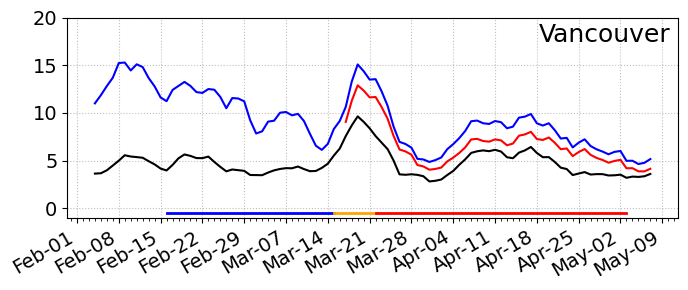

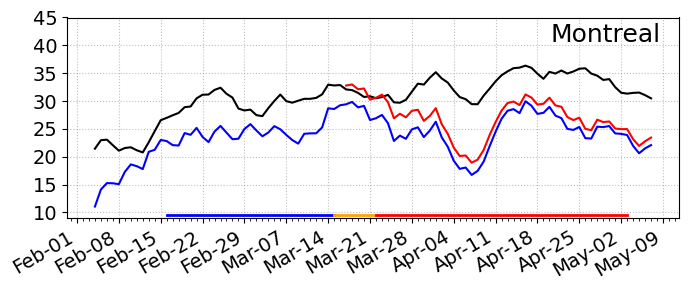

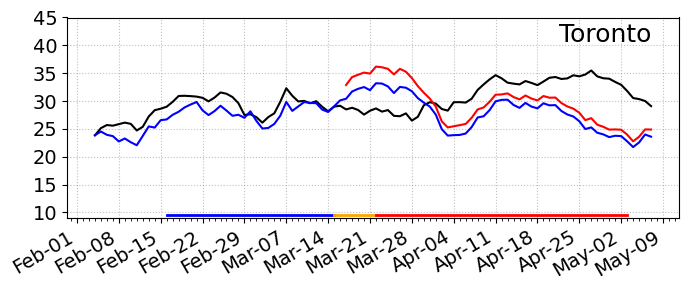

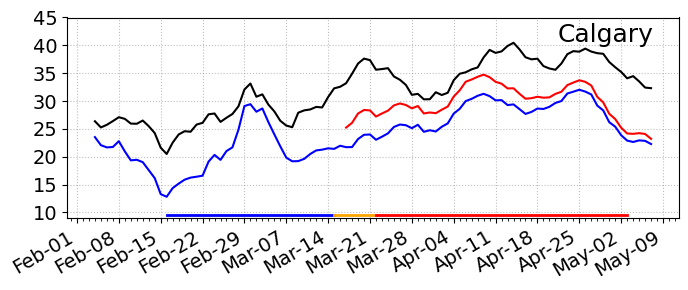

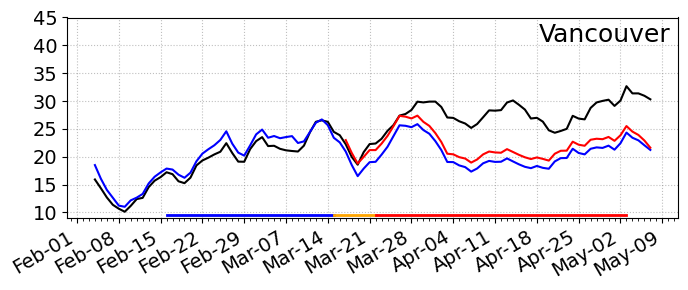

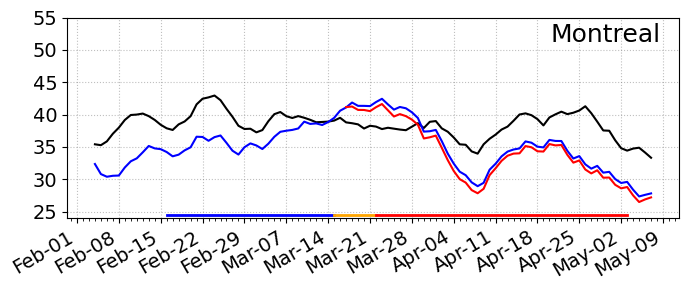

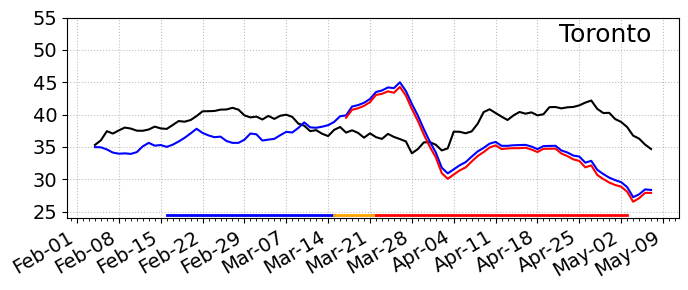

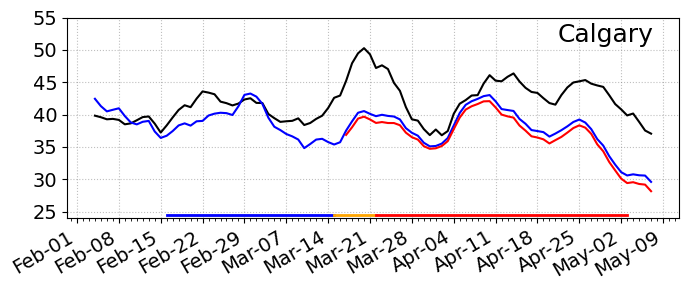

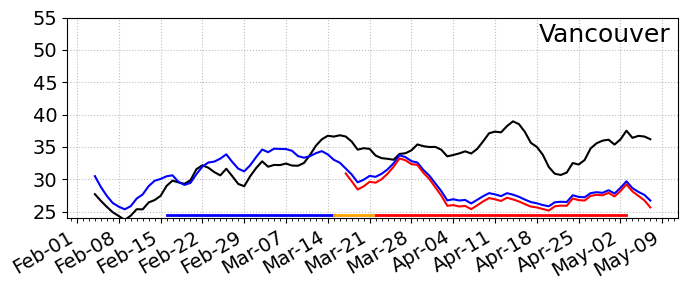


1. O_3_ (ppbv)
2. O_x_ (ppbv)

Figure S7. Time series of rolling 7-day averages of (a) NO_2_, (b) PM_2.5_, (c) O_3_, and (d) O_x_ (=O_3_+NO_2_) concentrations averaged over all monitoring stations within each metropolitan area for observations (black line) and for modelled values under the BAU (blue line) and COVID (red line) scenarios. The blue, yellow and red lines shown below each panel indicate the pre-lockdown period, transition to full lockdown, and the full lockdown period, respectively.

1. NO_2_ (ppb)


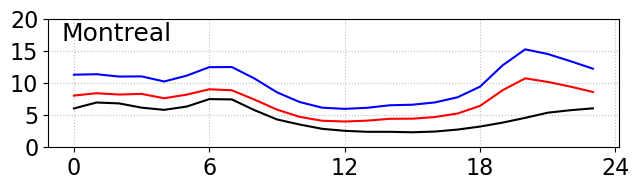

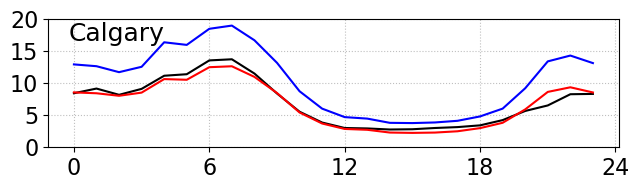

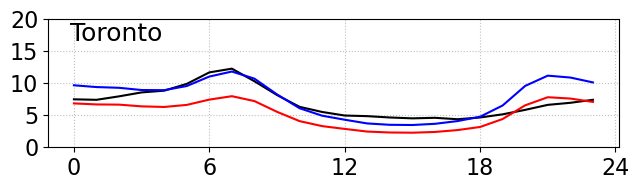

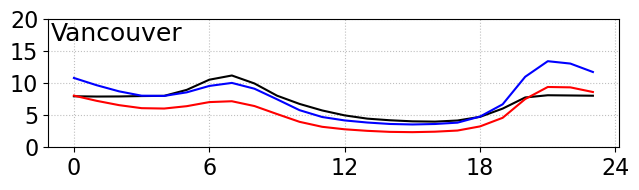


1. PM_2.5_ (µg m^-3^)


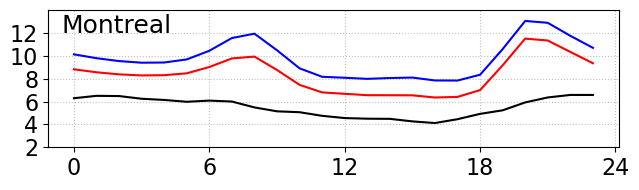

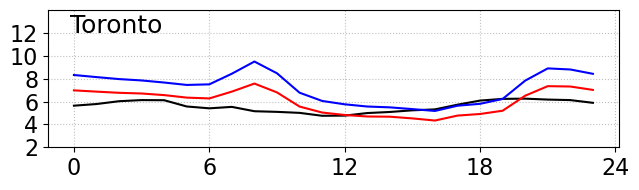

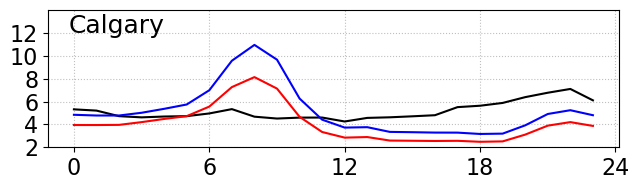

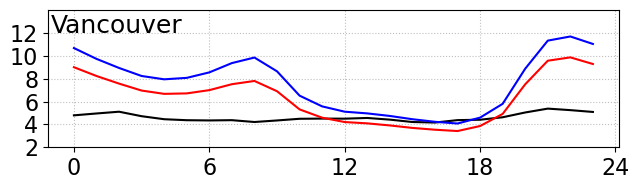


1. O_3_ (ppbv)


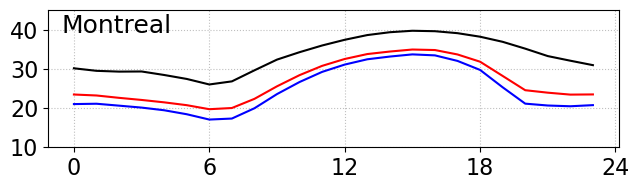

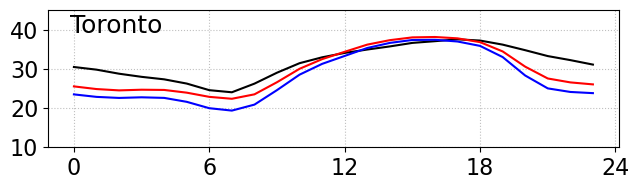

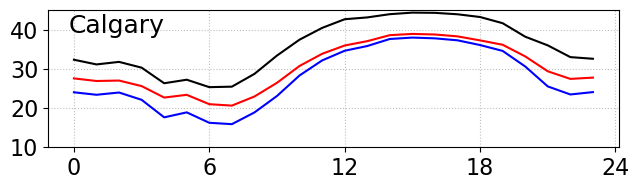

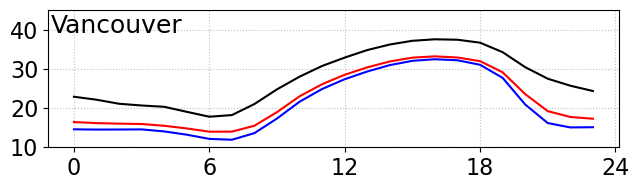


1. O_x_ (ppbv)


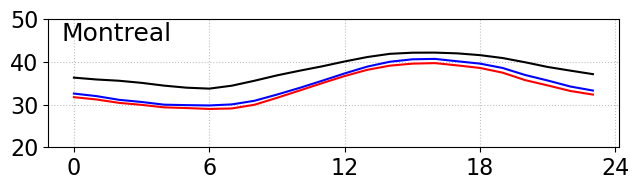

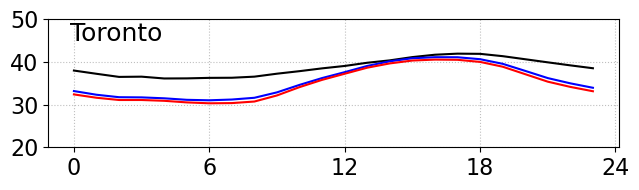

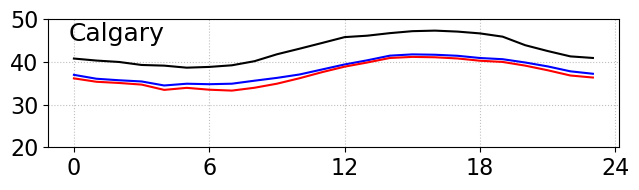

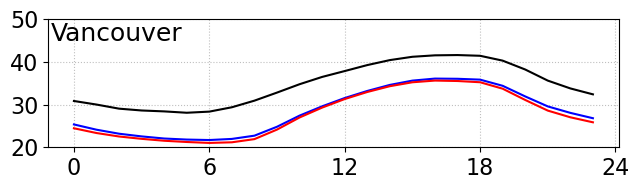


Figure S8. Mean diurnal time series of (a) NO_2_, (b) PM_2.5_, (c) O_3_, and (d) O_x_ (=O_3_+NO_2_) for the ‘full lockdown’ period averaged over all monitoring stations within each metropolitan area for observed concentrations (black line) and for predicted concentrations under the BAU (blue line) and COVID (red line) scenarios.

| 1. BAU | 1. COVID | (c) BAU-COVID | (d) (BAU-COVID)/BAU | Improvement |
| --- | --- | --- | --- | --- |
| 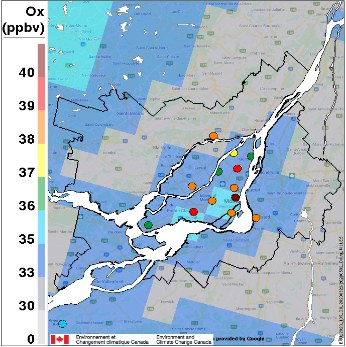  Montreal | 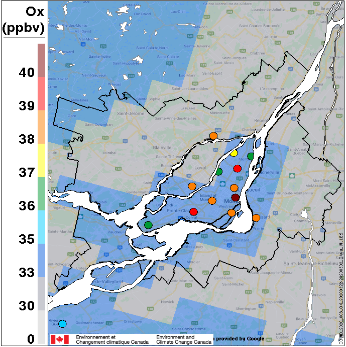 | 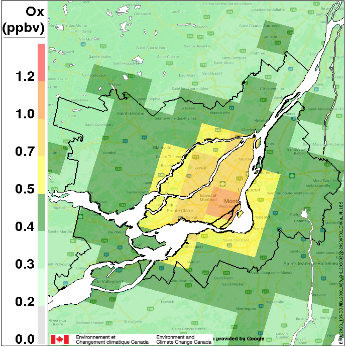 | 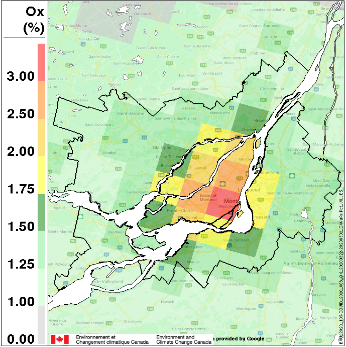 |  |
| 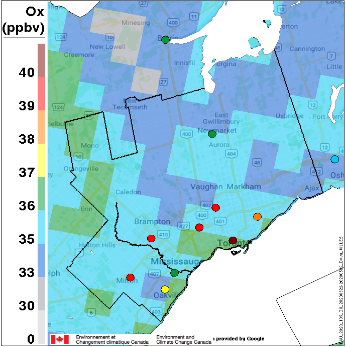  Toronto | 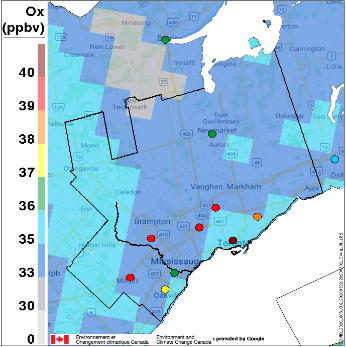 | 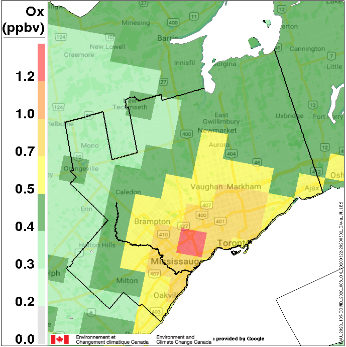 | 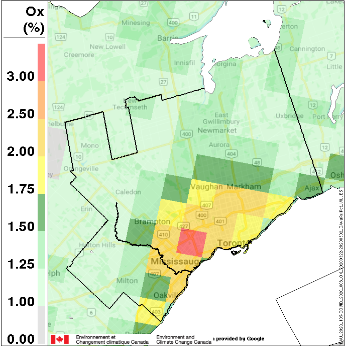 |  |
| 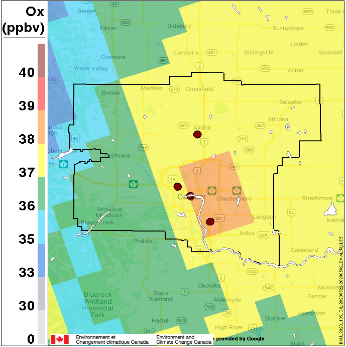  Calgary | 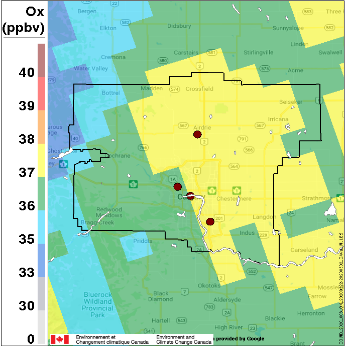 | 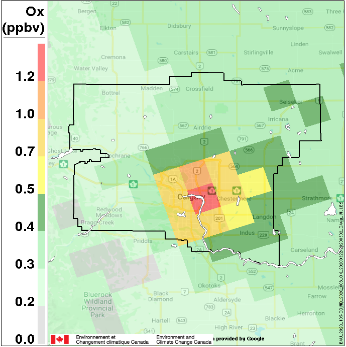 | 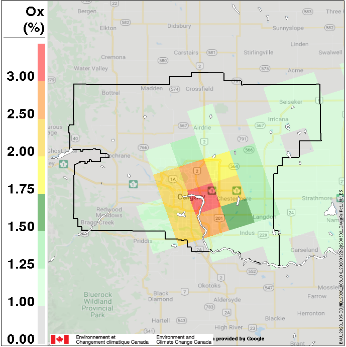 |  |
| 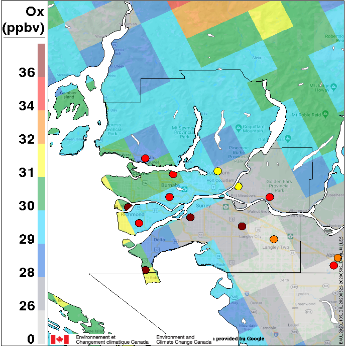  Vancouver | 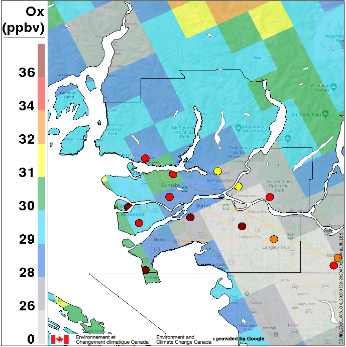 | 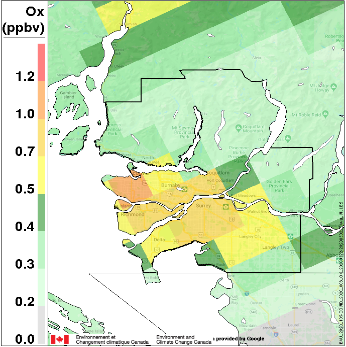 | 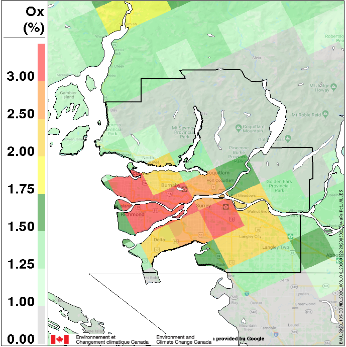 |  |

Figure S9. Mean hourly O_x_ (=O_3_+ NO_2_) surface volume mixing ratio (ppbv) gridded fields averaged over the ‘full lockdown’ period predicted by the model for the four major metropolitan areas for (a) BAU scenario, (b) lockdown scenario, (c) scenario difference (BAU – COVID), and (d) relative percentage difference ((BAU-COVID)/BAU). From top to bottom, the four rows correspond to Montreal, Toronto, Calgary, and Vancouver. Colored circles represent the location and the mean observed O_x_ VMR at each monitor and the thin black outlines indicate CMA boundaries.

Table S3. Mean hourly NO_2_, PM_2.5_, and O_3_ surface concentrations for the lockdown period averaged over all monitoring stations within each metropolitan area for observed concentrations and for model-predicted concentrations for the BAU and COVID-19 emissions scenarios for the four metropolitan areas. %Change is defined as (BAU – COVID-19) / BAU.

| **City** | **# Stations** | **Pollutant** | **Observed** | **BAU** | **COVID-19** | **%Change** |
| --- | --- | --- | --- | --- | --- | --- |
| **Montreal** | 14 | NO_2_ (ppbv) | 4.67 | 9.62 | 6.73 | 30.02 |
|  | 15 | PM_2.5_ (µg m^-3^) | 5.24 | 9.31 | 7.95 | 14.59 |
|  | 13 | O_3_ (ppbv) | 32.88 | 24.88 | 27.01 | -8.54 |
| **Toronto** | 9 | NO_2_ (ppbv) | 7.03 | 7.45 | 5.10 | 31.59 |
|  | 9 | PM_2.5_ (µg m^-3^) | 5.41 | 6.81 | 5.69 | 16.39 |
|  | 9 | O_3_ (ppbv) | 31.05 | 27.73 | 29.48 | -6.29 |
| **Calgary** | 4 | NO_2_ (ppbv) | 7.40 | 10.63 | 7.00 | 34.09 |
|  | 4 | PM_2.5_ (µg m^-3^) | 5.60 | 5.76 | 4.54 | 21.30 |
|  | 4 | O_3_ (ppbv) | 35.08 | 26.27 | 29.01 | -10.41 |
| **Vancouver** | 12 | NO_2_ (ppbv) | 7.92 | 7.88 | 5.64 | 38.37 |
|  | 11 | PM_2.5_ (µg m^-3^) | 4.96 | 7.92 | 6.60 | 16.72 |
|  | 12 | O_3_ (ppbv) | 27.09 | 20.65 | 22.17 | -7.37 |


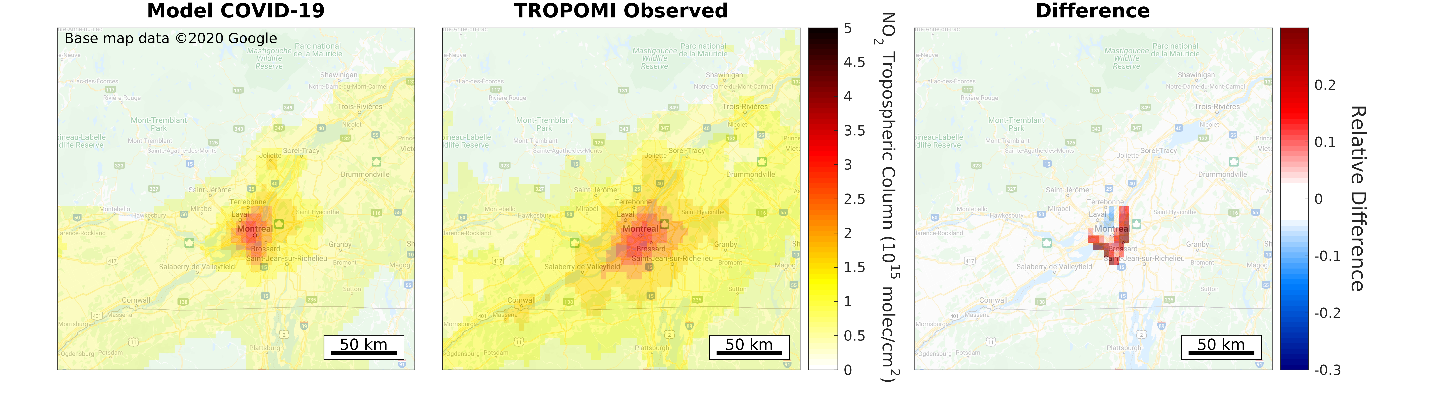


1. **(b) (c)**

Figure S10.  Mean NO_2_ VCDs from (a) model predictions for the COVID emissions scenario and (b) TROPOMI observations over the Montreal region of southern Quebec for the period 1 April – 8 May 2020.  Relative differences between the two fields [(observations-model)/model] are shown in panel (c) for areas that exceed 2 x10^15^ molec cm^-2^.


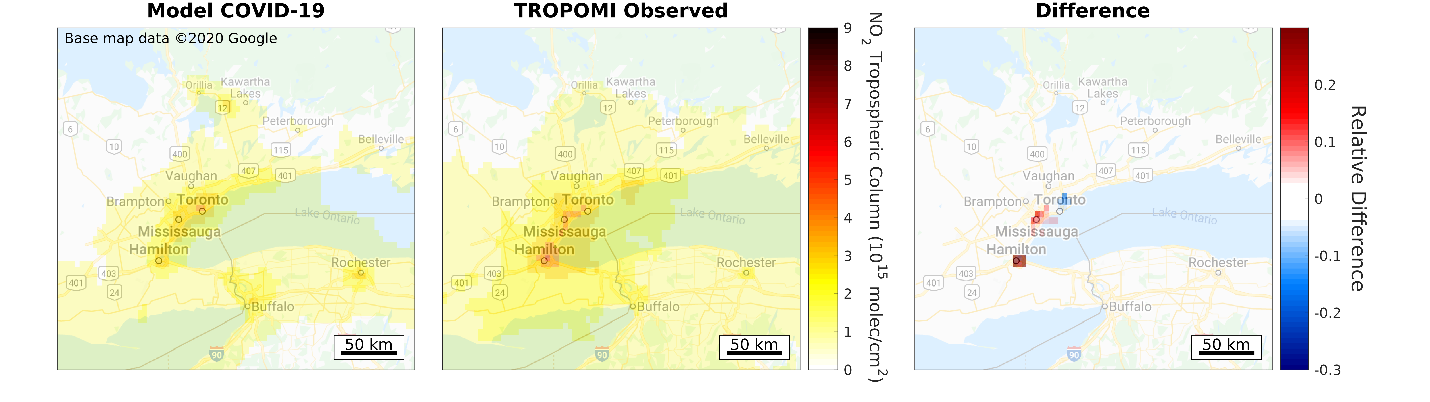


**(a) (b) (c)**

Figure S11.  Same as Figure S10 but for the Toronto region of southern Ontario.  The relative differences

[(observations-model)/model] are shown in panel (c) for areas that exceed 3 x10^15^ molec cm^-2^.
